# Scorpion venom peptides enhance immunity and survival in *Litopenaeus vannamei* through antibacterial action against *Vibrio parahaemolyticus*

**DOI:** 10.3389/fimmu.2025.1551816

**Published:** 2025-04-22

**Authors:** Ling Zeng, Yulin Sun, Hualin Zhang, Xiangxi Yi, Ran Du, Ziming Chen, Qi Wang

**Affiliations:** ^1^ School of Chemistry and Chemical Engineering, Lingnan Normal University, Zhanjiang, Guangdong, China; ^2^ Life Science & Technology School, Lingnan Normal University, Zhanjiang, Guangdong, China; ^3^ Guangxi Key Laboratory of Marine Drugs, Guangxi University of Chinese Medicine, Nanning, Guangxi, China; ^4^ Shenzhen Institute of Guangdong Ocean University, Guangdong Ocean University, Shenzhen, Guangdong, China

**Keywords:** scorpion venom peptide, BmKn1, *Litopenaeus vannamei*, immunity, *Vibrio parahaemolyticus*, antibacterial mechanism

## Abstract

**Introduction:**

Scorpion venom-derived antimicrobial peptides (AMPs) have emerged as promising candidates for combating bacterial infections owing to their potent activity and unique mechanisms of action. This study focuses on three 13-amino-acid peptides—BmKn1, BmKn2, and BmKn2-7—derived from the venom of *Mesobuthus martensii*. The aim is to elucidate their structural features, antibacterial efficacy, and immunomodulatory effects in *Litopenaeus vannamei* infected with *Vibrio parahaemolyticus* (VP).

**Methods:**

The peptides were synthesized and comprehensively characterized for their amphipathic α-helical structures, net charges, and hydrophobicity. Their antibacterial mechanisms were investigated using a series of assays, including membrane permeability (inner/outer membrane disruption), membrane depolarization, reactive oxygen species (ROS) quantification, and ATPase activity measurement. In vivo challenge experiments were conducted to evaluate survival rates in L. vannamei infected with VP. Additionally, immune enzyme activities (phenoloxidase [PO], complement component 3 [C3]) and inflammatory/antimicrobial gene expression levels (TNF-α, IL-1β, TGF-β, ALF, Crus) were analyzed. Furthermore, intestinal transcriptome profiling was performed to identify the activated immune pathways.

**Results:**

All peptides exhibited membrane-targeting activity: BmKn2-7 showed superior outer membrane penetration and depolarization, while BmKn1 was more effective in inner membrane disruption and ROS induction. In vivo, all peptides significantly improved survival rates in VP-infected shrimp (P < 0.01), with BmKn2-7 ≈ BmKn1 > BmKn2 in efficacy. Immune modulation was evident through increased PO and C3 activity (P < 0.05) and reduced expression of inflammatory cytokines and antimicrobial genes (P < 0.05). Transcriptome analysis revealed BmKn2-7 activated PPAR, AMPK, and FoxO signaling pathways.

**Discussion:**

The amphipathic α-helical structure of these peptides is fundamental to their membrane-disruptive activity. The enhanced outer membrane targeting of BmKn2-7 likely correlates with structural modifications that optimize hydrophobicity and charge distribution. The differential efficacy in immune regulation, such as BmKn2-7's broad pathway activation versus BmKn1's selective ROS induction, indicates structure-dependent functional divergence. These findings highlight the potential of tailored scorpion venom peptides as dual-action agents against bacterial infections and immune dysregulation

## 1 Introduction

*Litopenaeus vannamei* is recognized as one of the most nutritionally and economically significant shrimp species in aquaculture (1). However, the intensification and industrialization of aquaculture practices have resulted in environmental degradation and an increased incidence of diseases among farmed animals, significantly impeding the growth of the shrimp aquaculture industry (2). Vibrio species are recognized as principal pathogens responsible for infectious disease outbreaks in shrimp aquaculture, leading to significant economic losses for farmers due to the elevated mortality rates in intensive culture systems (3–6). In response to these diseases, large amounts of antibiotics are frequently employed. This excessive reliance on antibiotics has not only facilitated the emergence of antibiotic-resistant pathogens but has also caused environmental damage and raised concerns about food safety. As a result, there is a growing interest in exploring natural and environmentally friendly treatment options to protect aquaculture (7).

Antimicrobial peptides (AMPs) demonstrate a wide range of bactericidal activities, effectively suppressing the growth of diverse microbial organisms, encompassing both gram-positive and gram-negative bacteria, protozoa, fungi, and enveloped viruses (8, 9). The positive charge characteristic of AMPs facilitates their binding to the negatively charged microbial membranes. AMPs disrupt the structural integrity of microbial organisms cell membranes, thereby hindering the development of drug resistance (10). Consequently, these peptides are regarded as a promising alternative to traditional antibiotics. AMPs not only exhibit significant antibacterial activity but also potentiate the host’s immune response. The immunomodulatory properties of certain peptides have been effectively utilized in agricultural trials (11, 12), especially in aquaculture (13–15), yielding results that stimulate innate immune responses and significantly improve overall health. AMPs are anticipated to offer a sustainable, safe, and effective approach for preventing and controlling diseases in aquaculture. One such AMP derived from scorpion venom is BmKn2, which exhibits potent antimicrobial activity against both Gram-positive and Gram-negative bacteria due to its alpha-helical structure and C-terminal amidation. A chemically modified variant of BmKn2, BmKn2-7, has been shown to exhibit superior antibacterial activity compared to its precursor.

In our preliminary studies, we identified another peptide, BmKn1, from the venom of the scorpion *Buthus martensii*, which shares an 85% amino acid sequence similarity with BmKn2. However, the functional characterization of BmKn1 has yet to be elucidated. Previous studies indicate that BmKn2 exhibits more potent antimicrobial activity compared to IsCT, a peptide utilized in aquaculture to enhance the growth performance and intestinal immune function of juvenile grass carp (*Ctenopharyngodon idella*). However, the functions of BmKn2 in aquaculture species have not been thoroughly investigated. It remains to be determined whether BmKn2, BmKn2-7, and BmKn1 can similarly enhance the growth performance and intestinal immune function of aquaculture species, warranting further in-depth investigation. This study aims to investigate the effects of peptides BmKn1, BmKn2, and BmKn2-7 on antivibriosis mechanisms, as well as their impact on the growth performance and immune response of *L. vannamei*. The results are expected to provide valuable insights for enhancing the production of *L. vannamei* and improving disease resistance.

## 2 Materials and methods

### 2.1 Materials

*Vibrio parahaemolyticus* was purchased from Guangdong Microbial Culture Collection Centre (GDMCC). 2216E liquid medium was purchased from Qingdao Hope Bio-Technology Co., Ltd. Reactive Oxygen Species Assay Kit, N-phenylnaphthalen-1-amine (NPN), Propidium iodide (PI) and 3,3’-Dipropylthiadicarbocyanine iodide (DiSC3 (5)) were purchased from Shanghai Aladdin Biochemical Technology Co., Ltd. Alkaline phosphatase and adenosine triphosphatase were assayed using commercial kits (Nanjing Jiancheng Biological Engineering Institute, China).

### 2.2 Synthesis and characterization of peptides

The three peptides BmKn1, BmKn2 and BmK2-7 were synthesized with amidated C-termini by Sangon Biotech (Shanghai) Ltd., China. The purity (>98%) of three peptides utilized in the biological experiments was assessed using RP-HPLC at 220 nm. The analysis was conducted on a C18 column measuring 4.6 × 250 mm, with a flow rate of 1.0 mL min^−1^. A water/acetonitrile gradient containing 0.1% trifluoroacetic acid was employed to ensure precise determination of peptide purity. The synthesized peptides were stored at a temperature of -80°C until further evaluations were conducted.

The hydrophobicity of the peptides and their hydrophobic moment (μH) values were determined using the HeliQuest analysis website, available at http://heliquest.ipmc.cnrs.fr/cgi-bin/ComputParams.py. Helical wheel projections were performed using the online tool Helical Wheel Projections, accessible at http://rzlab.ucr.edu/scripts/wheel/wheel.cgi. To predict the three-dimensional structure of the peptides, I-TASSER was employed from http://zhanglab.ccmb.med.umich.edu/I-TASSER/.

### 2.3 Antimicrobial mechanism

#### 2.3.1 External membrane permeation test

The effects of BmKn1, BmKn2, and BmK2-7 on bacterial outer membrane permeability were assessed using a hydrophobic fluorescent probe. The VP strain was cultured in 2216E medium for 24 hours and subsequently centrifuged. The bacteria were washed with PBS and resuspended to an OD600 of 0.5. A final concentration of 40 μM NPN and 125 μg/mL of each of the three peptides were added to the bacterial suspension. Sterile PBS served as the control (CG). Following a 6-hour incubation at 37°C, the fluorescence intensity at 420 nm (excitation wavelength 350 nm) was measured using an MD SpectraMax i3x multifunctional enzyme labeller.

#### 2.3.2 Inner membrane permeability test

The effect of BmKn1, BmKn2, and BmK2-7 on the permeability of the VP inner membrane were assessed by PI determination. Bacteria cultured for 24 hours were harvested by centrifugation, washed with PBS, and resuspended to an OD600 of 0.5. PI was added to achieve a final concentration of 40 μM, in conjunction with 125 μg/mL of each of the three peptides. PBS was used as the control group. Following a 6-hour incubation at 37°C, the fluorescence intensity at 617 nm (excitation wavelength of 535 nm) was quantified using an MD SpectraMax i3x multifunctional enzyme labeller.

#### 2.3.3 Cytoplasmic membrane depolarization test

The depolarizing effects of BmKn1, BmKn2, and BmK2-7 on bacterial cytoplasmic membranes were assessed using the membrane potential-sensitive fluorescent dye DISC3 (5). Bacterial cultures, grown for 24 hours, were harvested via centrifugation, washed with PBS, and resuspended to an optical density (OD600) of 0.5. Subsequently, the bacterial suspension was incubated with a final concentration of 20 μM DISC3 (5) dye for 10 hours at 37°C in the dark. The three peptides (125 μg/mL) were subsequently added to the mixture, with sterile PBS used as the control. Following a 6-hour incubation period at 37°C, the fluorescence intensity at 670 nm (excitation wavelength 622 nm) was quantified using an MD SpectraMax i3x multifunctional enzyme labeller.

#### 2.3.4 Measurement of reactive oxygen species

Bacterial intracellular ROS levels were quantified using a ROS assay kit. The VP culture was incubated for 24 hours, after which the cells were harvested by centrifugation, washed with PBS, and resuspended to an optical density (OD600) of 0.5. Equal volumes of the bacterial suspension and the three peptide solutions (125 μg/mL each) were combined and incubated at 37°C for 10 hours. A final concentration of 10 μM DCFH-DA solution was then added. Sterile PBS served as the control group. Following a 6-hour incubation at 37°C, the fluorescence intensity at 525 nm (excitation wavelength 488 nm) was measured using an MD SpectraMax i3x multifunctional enzyme labeller.

#### 2.3.5 Alkaline phosphatase test

The VP was incubated for 24 hours, followed by centrifugation to collect the organisms, which were then washed with PBS and resuspended to an OD600 of 0.5. Subsequently, the three peptides were added sequentially, each at a final concentration of 62.5 μg/mL. A sterile PBS solution served as the control group. The samples were then incubated at 37°C for 10 hours, after which alkaline phosphatase activity was measured according to the kit instructions.

#### 2.3.6 Adenosine triphosphatase activity test

The VP was incubated for 24 hours, followed by centrifugation to collect the organisms. The collected organisms were washed with PBS and resuspended to an OD600 of 0.5. The three peptides were then added sequentially, each achieving a final concentration of 62.5 μg/mL. A sterile PBS solution served as the control group. The samples were then incubated at 37°C for 6 hours, after which adenosine triphosphatase activity was measured according to the kit instructions.

### 2.4 Challenge test

The challenge test for *L. vannamei* was conducted at the Marine Biology Research Base of Guangdong Ocean University in Zhanjiang, China. The *L. vannamei* specimens were sourced from Zhanjiang Hisenor Marine Biotechnology Co., Ltd., also located in Zhanjiang, China. VP for the challenge test was obtained from the Guangdong Microbial Culture Collection Centre (GDMCC 1.306). The strain was cultured in 2216E liquid medium at 37°C for 24 hours, followed by centrifugation. According to the preliminary experiment results, the centrifuged bacterial suspension was adjusted to a concentration of 5 × 106 CFU/ml using PBS. Additionally, the three peptides were prepared in PBS at a concentration of 125 μg/mL (use it right after it was ready). The challenge test was divided into five groups, each with three replicates, and each replicate comprised 30 shrimp (7.00 ± 0.50g). In the control group (CG), each shrimp received an injection of 100 µL of sterile physiological saline. In the negative control group (VA), each shrimp was first injected with 50 µL of VP, followed by an injection of 50 µL of sterile physiological saline 2 hours later. The experimental groups (Bmkn1, Bmkn2, and Bmkn2-7) were injected with three different types of scorpion venom peptides, respectively. Specifically, each shrimp was first injected with 50uL of VP, and then 2h later an equal volume of scorpion venom peptide was injected separately. The mortality rate was recorded every 24 hours post-injection, and the challenge test continued for 7 days.

#### 2.4.1 Immune response of *L. vannamei*

At the end of the challenge test, three shrimp hepatopancreases were randomly selected from each replicate and stored at -80°C for enzyme activity analysis. Additionally, three shrimp hepatopancreases were collected from each replicate and preserved in 1.5 mL RNA Later solution in enzyme-free centrifuge tubes for gene expression analysis. The levels of hepatopancreatic phenoloxidase (PO, H247-1-2), malondialdehyde (MDA, A003-1-2), lysozyme (LZM, A050-1-1), complement 3 (C3, H186-1-2), and complement 4 (C4, H186-2-2) were measured following the protocols provided by commercial kits (Nanjing Jiancheng Biological Engineering Institute, China).

The total RNA from the samples was extracted using an RNA extraction kit (Hunan Aikeri Biological Engineering Co., Ltd., Hunan, China). The RNA concentration was quantified using a Nanodrop2000 nucleic acid protein analyzer (Thermo Scientific), and the quality was evaluated by 1% agarose gel electrophoresis. RNA was reverse transcribed into cDNA using the Evo M-MLV RT Kit with gDNA Clean for qPCR II (Hunan Aikeri Biological Engineering Co., Ltd., Hunan, China). The primers listed in **Table 1** were synthesized by Shanghai Sangon Biotech Co., Ltd. Qualitative analysis of the cDNA was conducted using a SYBR reagent kit (Hunan Aikeri Biological Engineering Co., Ltd.). A 10 μL reaction mixture was prepared, consisting of 5 μL SYBR^®^ Green Pro Taq HS, 1 μL cDNA, 0.5 μL of each primer (forward and reverse), and 3 μL nuclease-free water. The reaction was performed on a quantitative thermal cycler under the following conditions: initial denaturation at 95°C for 30 seconds; 40 cycles of denaturation at 95°C for 5 seconds, annealing at 60°C for 30 seconds; and a final melt curve analysis at 95°C, followed by cooling to 4°C. The relative gene expression levels were determined using the 2^-ΔΔCT^ method.

**Table 1:** Primers used in this experiment for quantitative RT-PCR.

Genes	Forward (5’-3’)	Reverse (5’-3’)	GenBank no.
*β-actin*	TGGACTTCGAGCAGGAGATG	GGAATGAGGGCTGGAACAGG	XM_027364954.1
*TNF-α*	CTCAGCCATCTCCTTCTTG	TGTTCTCCTCGTTCTTCAC	XM_027368774.1
*IL-1β*	TGTGACCACCATCCACCAGAAC	GATCCCGCAGTAACCGAATAAG	Designed by author
*TGF-β*	GAAGCAATAAACCAAAGCGA	CAAAAGCCAACAGGGAAAA	XM_027378574.1
*ALF*	CGCTTCACCGTCAAACCTTAC	GCCACCGCTTAGCATCTTGTT	KJ000049
*Pen-3*	ATACCCAGGCCACCACCCTT	TGACAGCAACGCCCTAACC	Y14926
*Crus*	GGTGTTGGTGGTGGTTTCCC	CGAGGCCAGCACACTTGTAG	AY486426
*Cyt-c*	AGGGAAAGAAGCTGTTCGTG	CAGTCGCTTGTGCCAGTTCC	KF601549.1
*Bax*	GGTGGAATCACAAGAGAGCGA	TGTTCTCCACGGTGTCTCAC	XM_027383277.1
*Bcl-2*	CCTTGCTTGACACAGTCGGA	CAGACAAGGTCGTGAGGTGG	XM_070143368.1
*Caspase3*	ACATTTCTGGGCGGAACACC	GTGACACCCGTGCTTGTACA	KC660103.1
*Caspase8*	CACGGAAGCTCTCCCTACAG	GAAGACCTTGGGTTTCCCCC	Designed by author
*P53*	CGAATCCCCACATCCACG	GGCGGCTGATACACCACC	KX179650.1
*Apod*	TCTTAACTGCTGCCCTCGTG	AAAGCTGTTGGAGAGAAGGGG	Primer design based on RNA-seq gene fragments.
*4CL1*	TGCATGTGGTGCTGTATATTGT	CGCTCGTGCTGATGTCCTAT	
*tpi1b*	CAAGGTCAGCCTCTTCCTCA	ACTTCATCTGGCCGAACAGG	
*GNBP1*	CAGCTCAGCTAACCAAGGCT	GGCTCGTCAGGAATTGGGAA	
*RBBP6*	GTGCGACCTCTGTTGTCTGA	ACTATTCCCCCTGAGTGGTCA	
*Hirip3*	TCAACGTCCCTCTGCTTTCA	CTCATCATGCGCCTTCTTGC	
*PLAT*	GTGGAGAAGAACGGCGACAT	CCTGTAACGAGTTGCCAGGT	
*SPE*	GCTGGTTAACGGACAGGTGA	CGGAGGTTGACGTCGTGATA	
*lbp-6*	AGAGAGGGAATGACAGTGCG	ATGGCGGCTATGCTCTTACG	
*PCK2*	GCTTCACCACAGACGCAAAG	CCTCCACATCACTTACCTTCAGA	
*nas-4*	TGACATCGGCCTCAGAATCG	TGTCAATGACTGCCCACGAA	
*SLC5A6*	CTATGACGGCGCTACTCTGG	CCCTCCACATCACTTACCTTC	

#### 2.4.2 Transcriptome sequencing

The transcriptomes of the intestines of *Litopenaeus vannamei* were sequenced in this study for the CG, VP, and BmK2-7 groups. At the end of the challenge test, intestines from three randomly selected shrimp from each replicate were collected and placed in 1.5 mL enzyme-free centrifuge tubes containing RNA Later for transcriptome sequencing. The experimental procedure is as follows: mRNA was enriched using mRNA Capture Beads, and after bead-based purification, the mRNA was fragmented at high temperatures. The fragmented mRNA served as the template for synthesizing the first-strand cDNA in a reverse transcriptase reaction system. During the synthesis of the second-strand cDNA, end repair and A-tailing were simultaneously performed. Subsequently, adapters were ligated, and target fragments were purified and selected using Hieff NGS^®^ DNA Selection Beads. Finally, PCR amplification was carried out to construct the sequencing library, which was analyzed using the Illumina NovaSeq X Plus platform. The sequenced data underwent quality control, sequence alignment analysis, and gene analysis. Principal Component Analysis (PCA) was performed using R (http://www.r-project.org/) to investigate the distance relationships between samples through dimensionality reduction. The input data for differential gene expression analysis were the read count data obtained from gene expression level analysis. EdgeR software was used for analysis, including GO and KEGG enrichment analysis. In this study, 12 DEGs were randomly selected for RT-qPCR to validate the results of RNA sequencing. The validation procedure was performed as described in section 2.4.1. For detailed procedures of transcriptome sequencing, refer to **Supplementary Material 1**.

### 2.5 Statistical analysis

The experimental data were presented as the mean ± standard error of the mean (Mean ± SEM) and were statistically analyzed using SPSS 23.0 software. One-way analysis of variance (ANOVA) was conducted, followed by Duncan’s multiple range test for *post-hoc* comparisons.

## 3 Results

### 3.1 Peptide characterization

**Figure 1:**
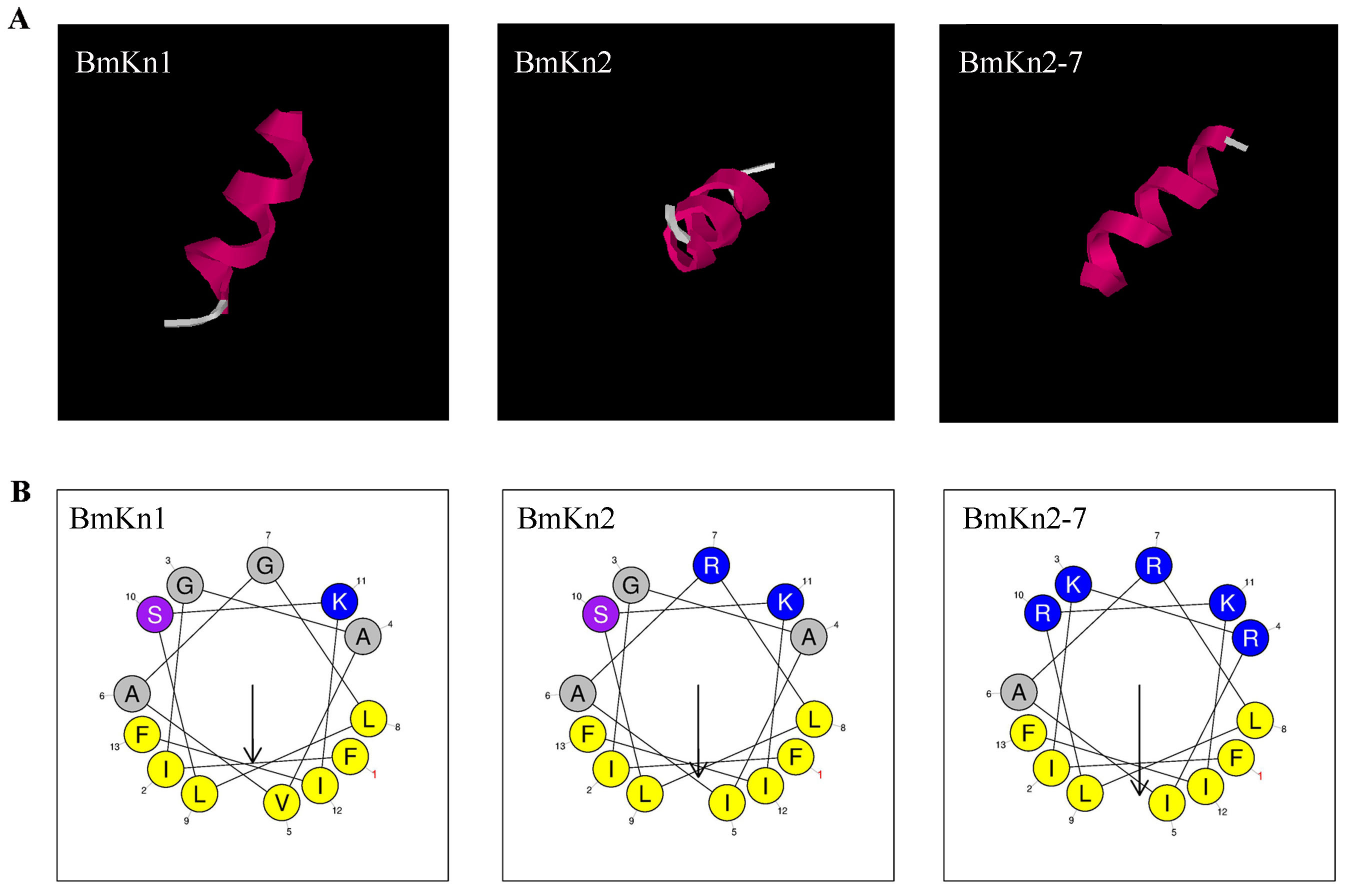
The structure of BmKn1, BmKn2 and BmKn2-7. **(A)** The prediction of three-dimensional structures of peptides utilizing I-TASSER. **(B)** Helical wheel projections of the three peptides. Alkaline amino acids, characterized by their positively charged residues, are highlighted in blue, whereas hydrophobic residues are illustrated in yellow.

BmKn1, BmKn2, and BmKn2-7, each comprising 13 amino acids and derived from scorpion venom, display similar sequences. The 3D structure of BmKn1, BmKn2 and BmKn2-7 were predicted by software I-TASSER, as illustrated in **Figure 1A**. Structural predictions suggest that all the three peptides exhibit amphipathic alpha-helical conformations. Their net charges are +2, +3, and +6, respectively. In terms of hydrophobic moments, BmKn1 has the lowest value, BmKn2 has an intermediate value, and BmKn2-7 has the highest value (**Table 2**). The composition of amino acids is illustrated in **Figure 1B**. Helical wheel analysis has demonstrated that the arrangement of amino acids within the sequences constitutes an amphipathic structure, distinguished by two distinct regions: one side of the helix is primarily hydrophobic, comprising hydrophobic residues, whereas the opposing side is hydrophilic, featuring a concentration of charged amino acids. By comparing BmKn1 with BmKn2 and BmKn2-7, it was observed that BmKn1 exhibits identical hydrophilic regions on one side of its helical structure, similar to those of BmKn2 and BmKn2-7. However, on the opposite side, BmKn1 displays a decreased charge density relative to BmKn2 and BmKn2-7, indicating a lesser degree of amphipathicity.

**Table 2:** The sequences, charges, hydrophobicity and hydrophobic moments of the three peptides.

peptides	sequences	Net Charge	H	µH	Similarty
BmKn1	FIGAVAGLLSKIF-NH_2_	+2	0.876	0.639	100%
BmKn2	FIGAIARLLSKIF-NH_2_	+3	0.843	0.760	85%
BmKn2-7	FIKRIARLLRKIF-NH_2_	+6	0.591	0.908	62%

### 3.2 Antimicrobial mechanisms

#### 3.2.1 External membrane permeation test

**Figure 2:**
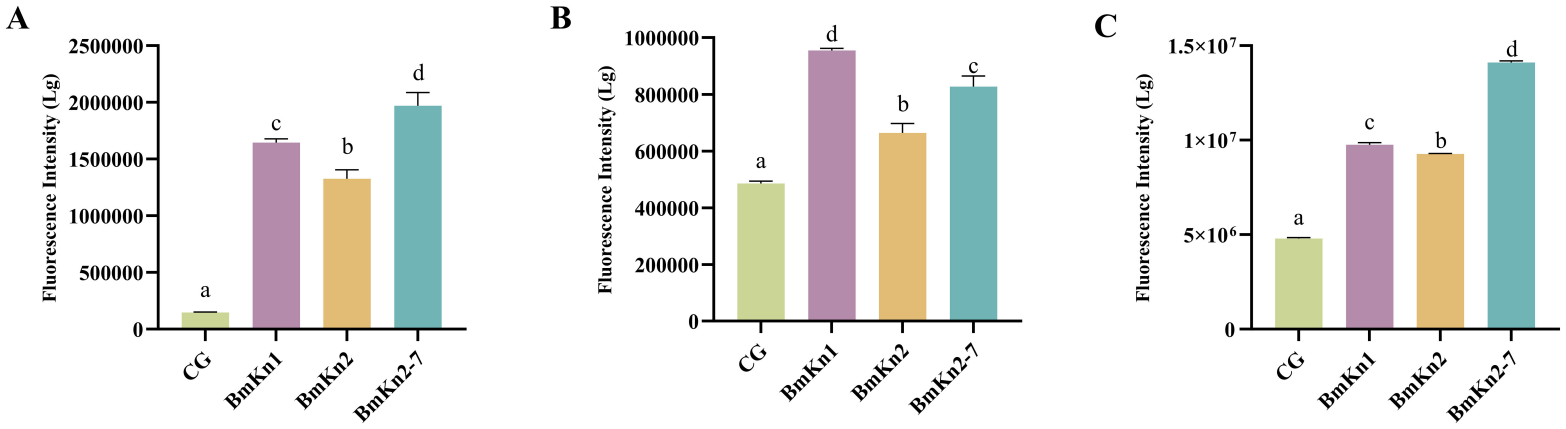
**(A)** Analysis of the external membrane permeability of VP in BmKn1, BmKn2, and BmKn2-7. **(B)** Analysis of inner membrane permeability of VP in BmKn1, BmKn2, and BmKn2-7. **(C)** Analysis of cytoplasmic membrane depolarization of VP in BmKn1, BmKn2, and BmKn2-7. Data was presented as mean ± SEM (n = 3). There were significant differences in the representation of different letters on the histogram (*P <*0.05).

The permeabilization of the outer membrane in VP after treatment with the three peptides was evaluated by monitoring the uptake of the fluorescent dye NPN, using an excitation wavelength of 350 nm and an emission wavelength of 420 nm. A significant increase in NPN fluorescence intensity was observed following the addition of the peptides (**Figure 2A**), suggesting that the peptides had compromised the integrity of the outer membranes. Upon comparing the fluorescence intensity of the three peptides, it was found that BmKn2-7 exhibits a higher value than both BmKn1 and BmKn2, with BmKn1 surpassing BmKn2. These results indicate that among the three peptides, BmKn2-7 possesses the strongest penetration capability, followed by BmKn1, while BmKn2 demonstrates the weakest penetration capability.

#### 3.2.2 Inner membrane permeability test

An extended analysis was conducted utilizing PI staining to assess the integrity of bacterial inner membranes. The fluorescence intensity observed in the experimental groups treated with the three peptides showed a significant increase, indicating compromised bacterial inner membranes post-peptide treatment (**Figure 2B**). Notably, among these groups, BmKn1 exhibited the most pronounced effect, with its fluorescence level being twice that of the control group. BmKn2-7 demonstrated a less pronounced yet still significant impact, whereas BmKn2 had the least effect, thus establishing the relative efficacy in disrupting the inner membrane as follows: BmKn1 > BmKn2-7 > BmKn2.

#### 3.2.3 Cytoplasmic membrane depolarisation test

To assess the effects of BmKn1, BmKn2, and BmKn2-7 on the cytoplasmic membrane of VP, the membrane potential-sensitive fluorescent dye diSC3 (5) was employed. Compared to the control group, the introduction of these peptides resulted in a significant increase in fluorescence intensity (**Figure 2C**), with the intensity surpassing that of the control by more than 100%. In the DiSC3 (5) assay, BmKn2 exhibited relatively diminished depolarizing activity on the bacterial cytoplasmic membrane compared to BmKn1 and BmKn2-7. Notably, BmKn2-7 achieved the highest fluorescence intensity, approximately tripling that of the control. Therefore, the depolarizing capabilities of the three peptides can be ranked as follows: BmKn2-7 > BmKn1 > BmKn2.

#### 3.2.4 Determination of ROS

**Figure 3:**
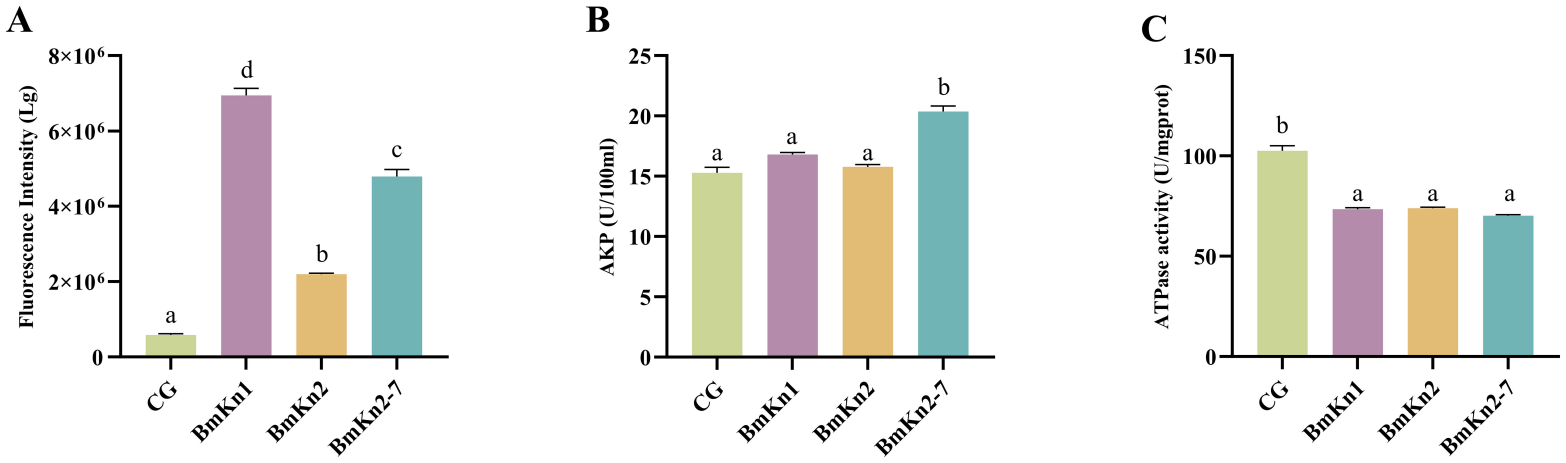
**(A)** Analysis of ROS of VP in BmKn1, BmKn2, and BmKn2-7. **(B)** Analysis of alkaline phosphatase activity of VP in BmKn1, BmKn2, and BmKn2-7 Variants. **(C)** Analysis of the ATPase activity of VP in BmKn1, BmKn2, and BmKn2-7. Data was presented as mean ± SEM (n = 3). There were significant differences in the representation of different letters on the histogram (*P <*0.05).

ROS are increasingly recognized for their critical role in bacterial responses to lethal stress. To assess the impact of BmKn1, BmKn2, and BmKn2-7 on intracellular ROS levels, the fluorescence intensity of DCFH-DA within the VP was utilized as an analytical indicator. Following the treatment of the three peptides, a significant elevation in ROS levels in the bacterial was observed, with levels increasing by at least 2.5-fold compared to the control group (**Figure 3A**). Notably, there were marked differences among the three experimental groups. Specifically, the ROS level induced by BmKn1 was more than three times higher than that of BmKn2, while the ROS level elicited by BmKn2-7 was over two times greater than that of BmKn2. These results suggest that all three peptides effectively induce a substantial increase in ROS levels in VP, with the order of efficacy being BmKn1 > BmKn2-7 > BmKn2.

#### 3.2.5 Alkaline phosphatase test

The impact of BmKn1, BmKn2, and BmKn2-7 on the enzymatic activity of VP alkaline phosphatase was evaluated. The findings indicated that the alkaline phosphatase activity in the experimental groups treated with BmKn1 and BmKn2 was marginally elevated compared to the control group; however, this increase was not statistically significant. Conversely, the alkaline phosphatase activity in the bacterial samples treated with BmKn2-7 demonstrated a substantial enhancement (**Figure 3B**). Following treatment with these three peptides, the relative alkaline phosphatase activities of VP were ranked in the following order: BmKn2-7 > BmKn1 > BmKn2.

#### 3.2.6 Adenosine triphosphatase activity test

To evaluate the impact of BmKn1, BmKn2, and BmKn2-7 on bacterial energy metabolism, the cellular ATPase activity activity of VP was assessed. The results indicated that there were no significant differences among the three peptides; however, they markedly reduced the cellular ATPase activity in VP by approximately 25% compared to the control (**Figure 3C**). These findings indicate that BmKn1, BmKn2, and BmKn2-7 can influence the energy metabolism of VP by decreasing ATPase activity.

### 3.3 Challenge test

**Figure 4:**
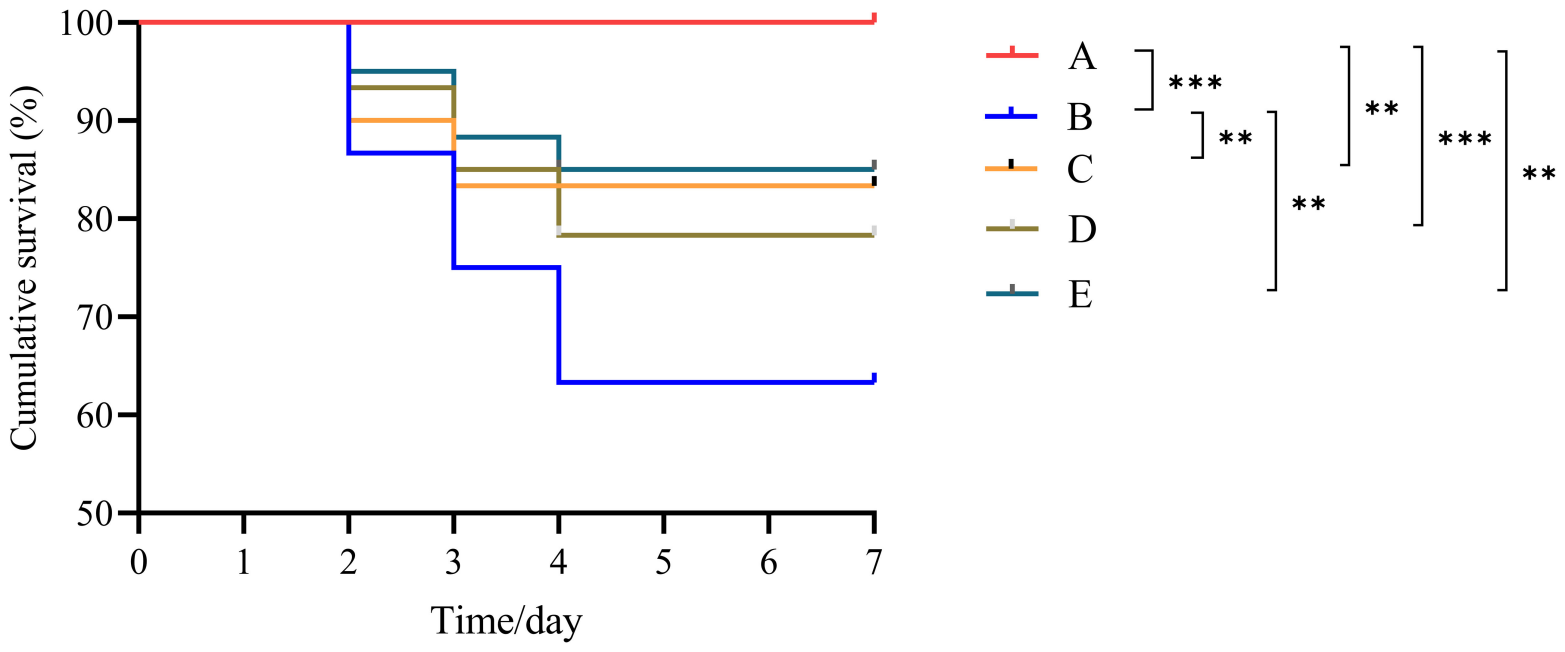
Cumulative survival of *L. vannamei* after challenge testing. ** indicates *P* < 0.01; *** indicates *P* < 0.001. A: CG; B: VP; C: BmKn1; D: BmKn2; E: BmKn2-7.

The challenge test results for *L. vanname*i are presented in **Figure 4**. No mortality was observed in the control group (CG). The cumulative survival rate of the VP group was significantly lower than that of all other groups (*P* < 0.05). Among the three peptide injection groups, no significant differences in cumulative survival rates were observed (*P* > 0.05); however, their survival rates were significantly lower than that of the CG (*P* < 0.05). Specifically, the survival rates among the peptide groups followed this order: BmKn2-7 > BmKn1 > BmKn2.

#### 3.3.1 Hepatopancreatic immune enzyme analysis

Hepatopancreatic immune enzyme activities in different treatment groups following challenge testing are summarized in **Table 3**. The VP group demonstrated significantly lower PO activity compared to the other groups (*P* < 0.05). LZM activity was lowest in the BmKn1 group and highest in the CG group (*P* < 0.05). C3 content was lowest in the VP group, while C4 content in the peptide supplementation groups was significantly lower than that in both the CG and VP groups (*P* < 0.05).

**Table 3:** Hepatopancreatic immune enzyme activity in different treatment groups after challenge testing.

Items	Treatment				
	CG	VP	BmKn1	BmKn2	BmKn2-7
PO (U/ml)	25.47+1.83^b^	21.34+1.15^a^	27.25+1.51^b^	26.15+0.69^b^	28.32+1.07^b^
LZM (U/L)	7.41+0.24^c^	5.23+0.09^b^	4.41+0.24^a^	5.32+0.27^b^	5.25+0.19^b^
C3 (μg/mL)	159.26+6.27^c^	79.08+2.35^a^	103.49+6.65^b^	103.40+4.67^b^	109.27+3.04^b^
C4 (μg/mL)	176.13+4.23^b^	208.88+2.45^c^	144.21+6.55^a^	141.29+5.62^a^	154.29+3.24^a^

Data was presented as mean ± SEM (n = 3). Different superscript letters represent significant differences (*P*<0.05).

#### 3.3.2 Hepatopancreatic immunity-related gene expression

The relative expression levels of *TNF-α* and *ALF* genes in the VP group were significantly higher than those in the other groups (*P* < 0.05; **Table 4**). Conversely, the relative expression of *IL-1*β gene was significantly lower in the BmKn1 and BmKn2-7 groups than in the other groups (*P* < 0.05). Additionally, the relative expression level of the *TGF-β* and *Bal-2* genes of CG was significantly higher than that in the VP and the three peptides injection groups (*P* < 0.05). There was no significant difference in the relative expression of *Cyt-c* gene between the groups (*P* > 0.05). Notably, the VP group exhibited the highest relative expression level of the Crus gene (P < 0.05). In contrast, the Pen-3 gene showed the lowest relative expression in the VP group, which significantly increased following peptide injection (*P* < 0.05). Furthermore, the relative expression of *Bax* gene in the peptide injection groups was significantly lower than that in the CG and VP groups (*P* < 0.05). Lastly, the relative expression levels of *Caspase3*, *Caspase8*, and *P53* genes were higher in the VP group compared to the peptide injection groups (*P* < 0.05).

**Table 4:** Relative expression of hepatopancreatic immunity genes in different treatment groups after challenge testing.

Items	Treatment				
	CG	VP	BmKn1	BmKn2	BmKn2-7
*TNF-α*	1.01+0.08^a^	1.54+0.06^b^	0.91+0.08^a^	0.92+0.11^a^	0.85+0.15^a^
*IL-1β*	1.00+0.03^c^	1.03+0.02^c^	0.64+0.01^a^	0.80+0.03^b^	0.63+0.04^a^
*TGF-β*	1.00+0.04^d^	0.65+0.05^bc^	0.46+0.04^a^	0.72+0.06^c^	0.51+0.08^ab^
*ALF*	1.01+0.02^a^	2.95+0.09^b^	0.95+0.07^a^	0.98+0.05^a^	0.99+0.03^a^
*Cyt-c*	1.00+0.03^a^	1.38+0.12^a^	1.21+0.20^a^	1.19+0.14^a^	1.07+0.06^a^
*Crus*	1.01+0.08^a^	1.89+0.08^c^	1.26+0.06^b^	1.44+0.03^b^	1.25+0.10^b^
*Pen-3*	1.00+0.03^d^	0.29+0.00^a^	0.54+0.02^b^	0.62+0.03^b^	0.82+0.04^c^
*Bax*	1.00+0.05^c^	1.01+0.04^c^	0.54+0.03^ab^	0.73+0.12^b^	0.41+0.05^a^
*Bcl-2*	1.01+0.10^b^	0.57+0.02^a^	0.72+0.03^a^	0.62+0.08^a^	0.63+0.02^a^
*Caspase3*	1.00+0.00^b^	1.84+0.09^c^	0.72+0.03^a^	0.73+0.07^a^	0.64+0.03^a^
*Caspase8*	1.00+0.04^b^	0.85+0.06^ab^	0.70+0.07^a^	0.74+0.06^a^	0.67+0.02^a^
*P53*	1.03+0.05^b^	1.29+0.02^c^	0.79+0.09^a^	0.72+0.05^a^	0.75+0.05^a^

Data was presented as mean ± SEM (n = 3). Different superscript letters represent significant differences (*P*<0.05).

#### 3.3.3 Transcriptome sequencing

**Figure 5:**
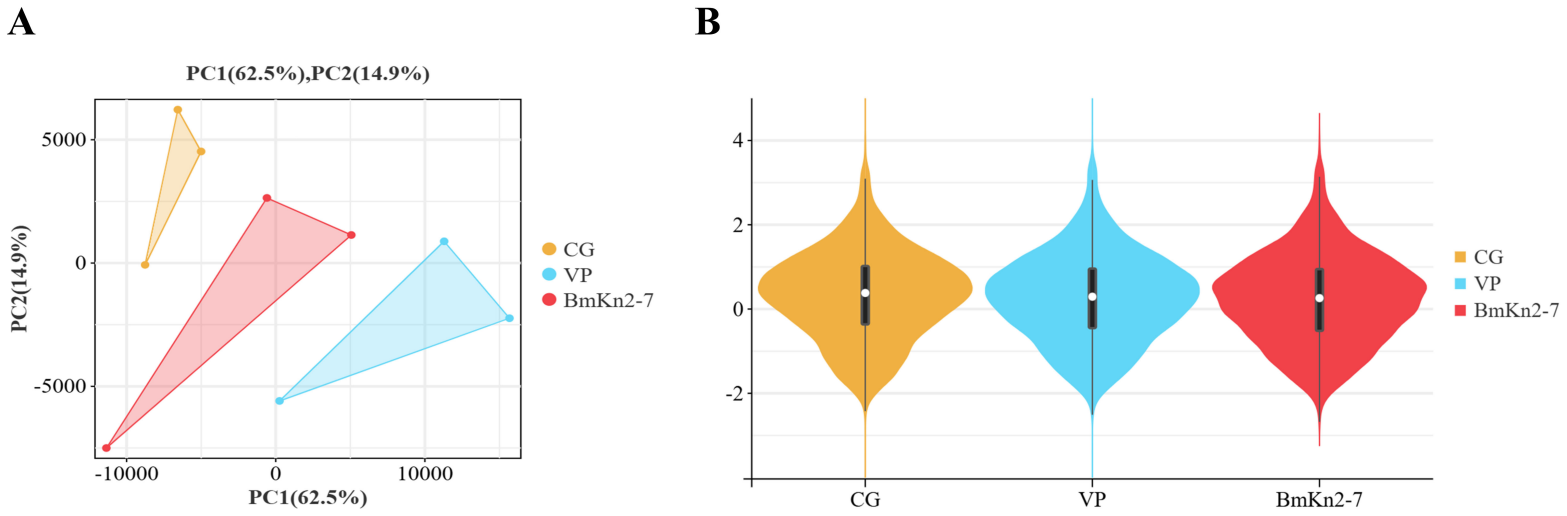
The relationship among transcriptome samples from the intestines of *Litopenaeus vannamei*. **(A)** PCA analysis of samples. **(B)** Violin plots of different groups.

**Figure 6:**
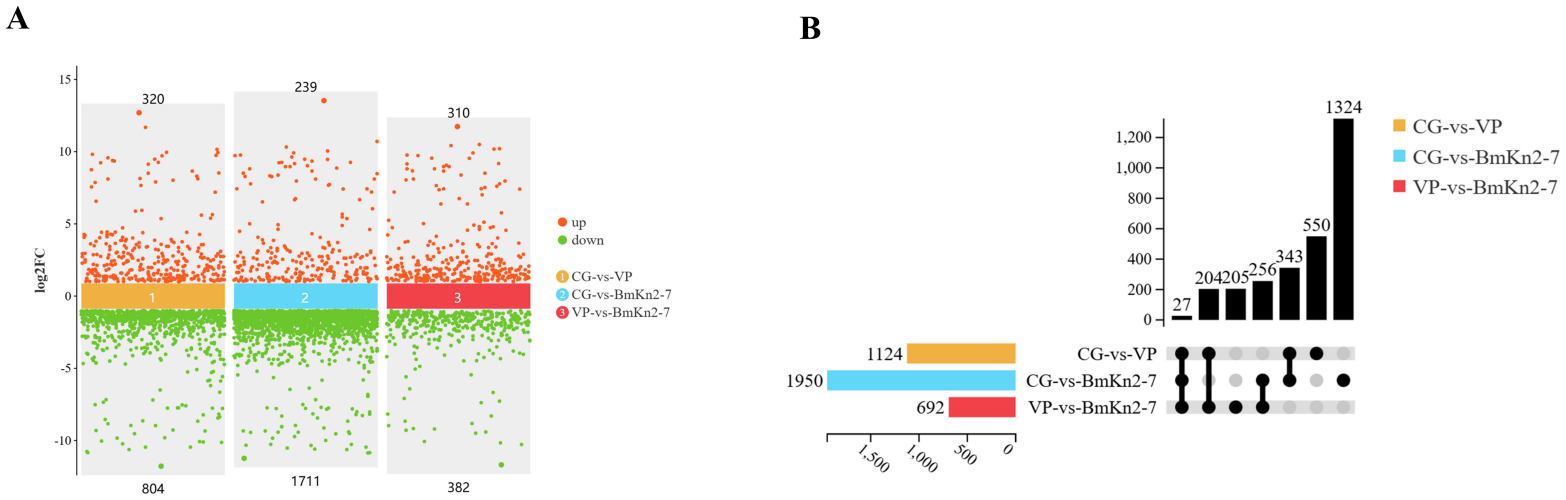
Differential analysis of the intestinal transcriptome of *Litopenaeus vannamei*. **(A)** Scatter plot of multiple group differences (*P* < 0.05, Fold change > 2). **(B)** Upset plot (*P* < 0.05, Fold change > 2).

The PCA analysis revealed that the CG, VP, and BmKn2-7 sample groups exhibit high levels of dispersion, indicating significant differences among the three groups (**Figure 5A**). The visualization of gene abundances across the three groups is shown in **Figure 5B**. The gene expression abundance in the VP and BmKn1 groups was slightly lower than that in the CG group. Further analysis was conducted to investigate the differences among various comparison groups. The scatter plot of multiple group differences is shown in **Figure 6A**. In the CG-vs-VP comparison group, 320 differentially expressed genes (DEGs) were upregulated, while 804 DEGs were downregulated. In the CG-vs-BmKn2-7 comparison group, 239 DEGs were upregulated, and 1711 DEGs were downregulated. In the VP-vs-BmKn2-7 comparison group, 310 DEGs were upregulated, and 382 DEGs were downregulated. The differences between comparison groups are visualized using upset plots, as shown in **Figure 6B**. The numbers of uniquely DEGs in the three comparison groups were 1324 (CG-vs-BmKn2-7), 550 (CG-vs-VP), and 205 (VP-vs-BmKn2-7), respectively.

**Figure 7:**
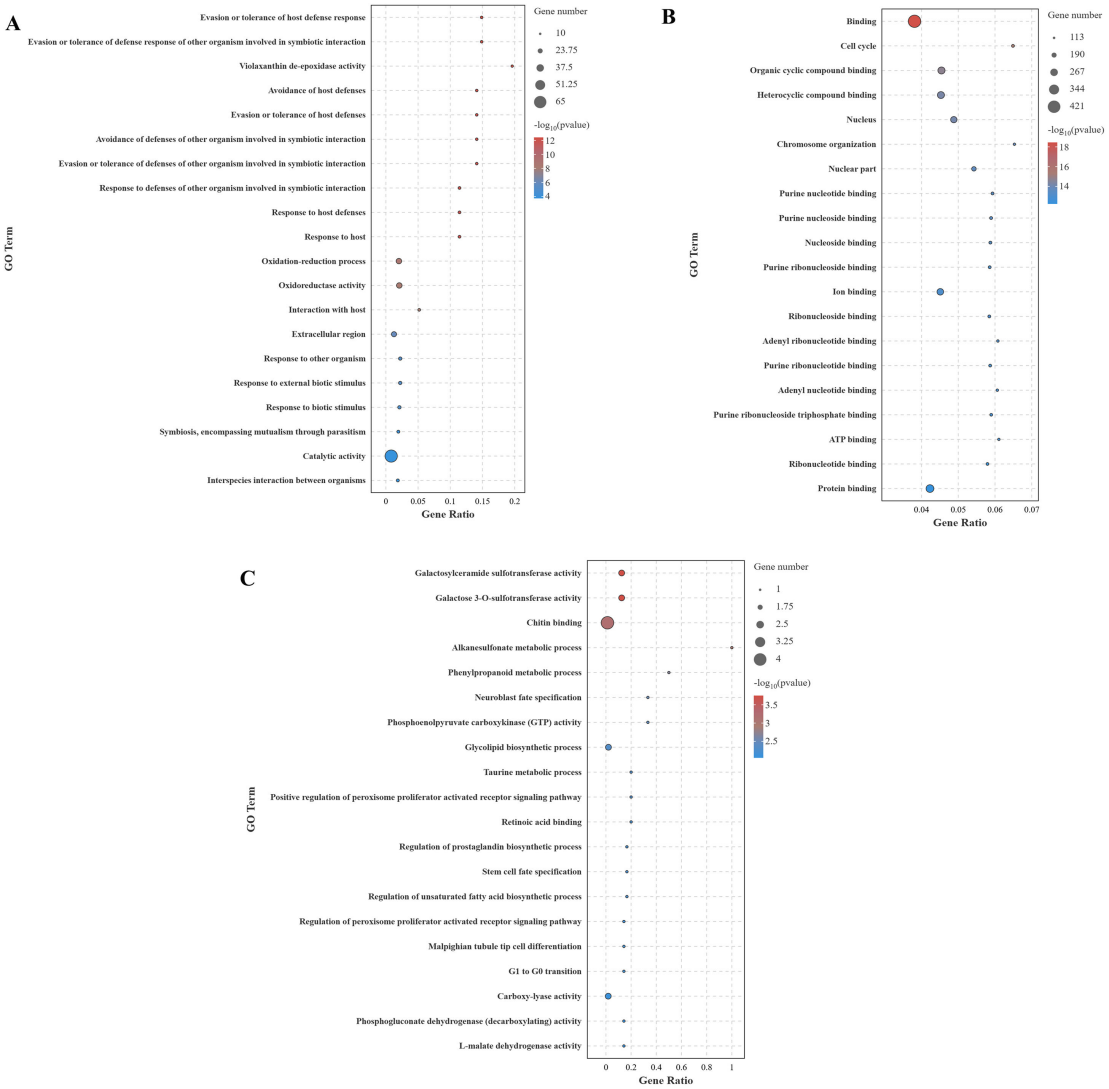
GO enrichment analysis of DEGs across different comparison groups (Top 20). **(A)** CG-vs-VP; **(B)** CG-vs-BmKn2-7; **(C)** VP-vs-BmKn2-7.

The GO enrichment analysis (top 20) of DEGs across different comparison groups is shown in **Figure 7**. In the CG-vs-VP comparison group (**Figure 7A**), the significantly enriched GO terms are predominantly related to immune responses, such as evasion or tolerance of host defense response, evasion or tolerance of defense response of other organism involved in symbiotic interaction, avoidance of host defenses, and avoidance of defenses of other organism involved in symbiotic interaction. Moreover, the DEGs annotated under these terms were all significantly downregulated. Similarly, the DEGs annotated under other GO terms were mostly significantly downregulated. And these DEGs are mainly *Apod* and *tpi1b*. Furthermore, it was found that most DEGs annotated to the term oxidoreductase activity were downregulated. In the CG-vs-BmKn2-7 comparison group, 16 of the top 20 GO terms were categorized under molecular function. GO terms with a higher number of annotated DEGs included binding, protein binding, organic cyclic compound binding, and heterocyclic compound binding (**Figure 7B**). In the VP-vs-BmKn2-7 comparison group, differential expression genes annotated with all terms, except for chitin binding, were significantly upregulated. The two most significantly enriched GO terms were galactosylceramide sulfotransferase activity and galactose 3-O-sulfotransferase activity. In addition, it was observed that the GO terms related to the PPAR signaling pathway, namely Positive regulation of peroxisome proliferator-activated receptor (PPAR) signaling pathway and Regulation of peroxisome proliferator-activated receptor signaling pathway, were significantly upregulated. The detailed information on the GO enrichment analysis of the three comparison groups is provided in **Supplementary Materials 2**.

**Figure 8:**
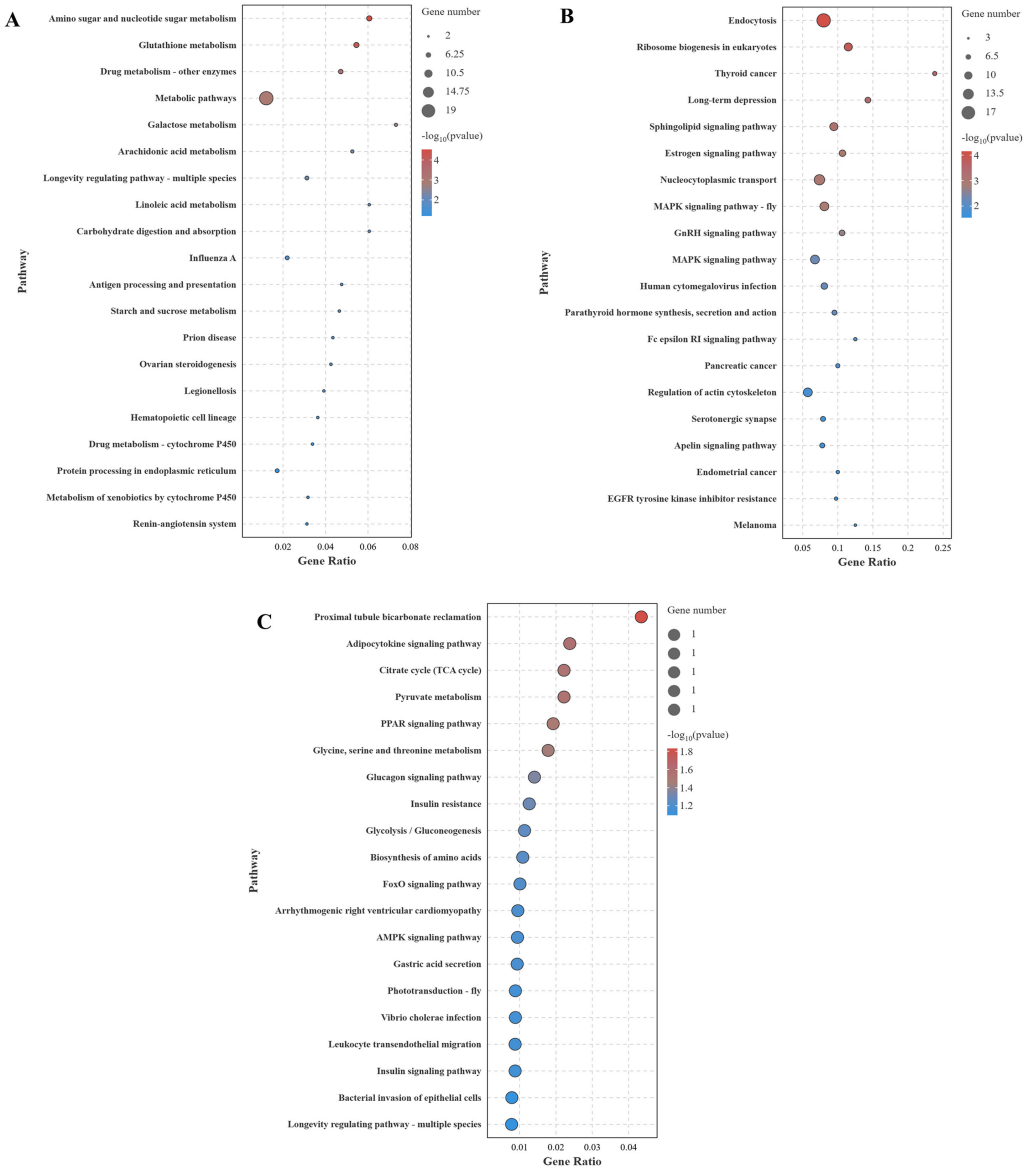
KEGG enrichment analysis of DEGs across different comparison groups (Top 20). **(A)** CG-vs-VP; **(B)** CG-vs-BmKn2-7; **(C)** VP-vs-BmKn2-7.

The KEGG enrichment analysis (top 20) of DEGs across different comparison groups is shown in **Figure 8**. In the CG-vs-VP comparison group, the largest number of DEGs were annotated to the Metabolic pathway, followed by Amino sugar and nucleotide sugar metabolism and Glutathione metabolism (**Figure 8A**). In addition, immune-related pathways were found to be significantly enriched, such as Influenza A, Antigen processing and presentation, and Hematopoietic cell lineage. In the CG-vs-BmKn2-7 comparison group, the largest number of DEGs was annotated to the Endocytosis pathway, followed by the MAPK signaling pathway, and Regulation of actin cytoskeleton (**Figure 8B**). In the VP-vs-BmKn2-7 comparison group (**Figure 8C**), Proximal tubule bicarbonate reclamation was the most significantly enriched, followed by the Adipocytokine signaling pathway and the Citrate cycle (TCA cycle). In addition, immune-related pathways were also significantly enriched, such as the PPAR signaling pathway, FoxO signaling pathway, and AMPK signaling pathway. Interestingly, the number of genes annotated to these pathways was limited to just one, with the key genes being *PCK2*. The detailed information on the KEGG enrichment analysis of the three comparison groups is provided in **Supplementary Materials 3**.

**Figure 9:**
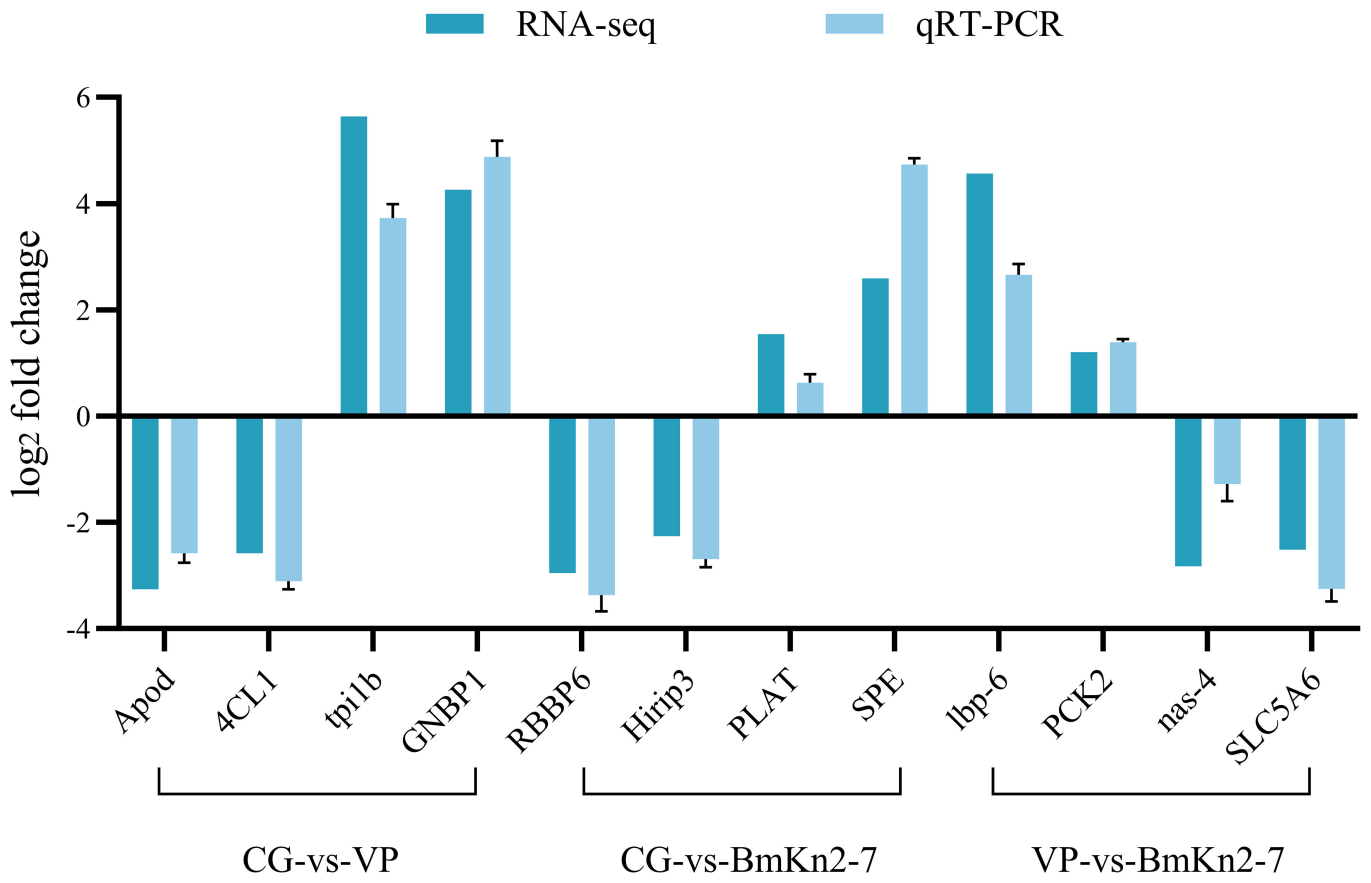
Validation of selected DEGs by qPCR.

The RT-qPCR results for the 12 randomly selected DEGs are shown in **Figure 9**. The expression levels of all 12 DEGs were consistent with the RNA-seq results, demonstrating the reliability of the RNA-seq data.

## 4 Discussion

The peptide BmKn1 was identified in the venom in our previous research (16), and its structure and function remains to be elucidated. BmKn1 shares a remarkably high homology (85%) with BmKn2. Both peptides originate from the venom of *Mesobuthus martensii*, have an identical length of 13 amino acids, and exhibit a high degree of sequence similarity, differing only at the fifth and seventh positions. The BmKn2-7 variant was developed from BmKn2 by enhancing the overall positive charge (17). The C-termini of all the three peptides have been amidated. Amidation of peptides is crucial for both their biological functions and resistance to exopeptidase degradation (18). Generally, C-terminally amidated peptides demonstrate enhanced structural stability and increased activity compared to their non-amidated counterparts (19). Secondary structure prediction by previoused research showed that BmKn2 contains one alpha-helix domain, and two flexible random coiled regions at both terminus (20). Our predicted results align with the aforementioned findings. Previous research on secondary structure prediction has demonstrated that BmKn-2 comprises one alpha-helix domain and two flexible random coil regions at its termini (20). Our predicted results align with this earlier finding. The secondary structures of the peptides BmKn1, BmKn2, and BmKn-7 display a high degree of similarity, characterized by the presence of a singular alpha-helical domain. Each peptide exhibits distinct hydrophobic and hydrophilic regions, which confer a pronounced amphipathic nature to their molecular structure.

The antimicrobial experiments demonstrated that BmKn1, BmKn2, and BmKn2-7 can eliminate bacteria through multiple mechanisms, including increasing the permeability of both inner and outer membranes, inducing cell membrane depolarization, elevating ROS levels, and reducing ATPase activity. These mechanisms collectively disrupt bacterial physiological functions. Notably, the antibacterial activities of these peptides exhibit significant variation, which can be attributed to their structural differences.

Charge and hydrophobicity are regarded as the primary physicochemical attributes that influence the biological activity of membrane-active peptides (21–23). Hydrophobicity governs the capacity of AMPs to partition into the lipid bilayer of microbial membranes. Positively charged AMPs will generate electrostatic attraction with the negatively charged components of the bacterial membranes. Enhancing the overall positive charge of AMPs can further strengthen this interaction (24). Research by Ringstad et al. (25) demonstrated that the capacity of peptides to compromise microbial membranes enhances as their net charge or hydrophobicity increase. This finding explains why BmKn1 exhibits superior external membrane penetration ability compared to BmKn2 in this study, as BmKn1 has a higher hydrophobicity (0.876 vs. 0.843). Additionally, BmKn2-7, which carries six positive charges, demonstrates the strongest external membrane penetration among the three peptides due to its higher number of positive charges compared to BmKn1 (two positive charges) and BmKn2 (three positive charges). However, our findings indicate that characterizing peptides solely by their hydrophobicity or charge is inadequate to fully elucidate their capacity to penetrate biological membranes. For instance, in this study, the hydrophobicity of the three peptides was ranked as BmKn1 > BmKn2 > BmKn2-7. Based on this criterion alone, one might expect a corresponding order of membrane penetration ability. Similarly, the charge distribution was observed as BmKn1 < BmKn2 < BmKn2-7, which would suggest a similar ranking for their penetration ability. However, contrary to these expectations, the experimental results demonstrated that the external membrane penetration ability of the three peptides was BmKn2-7 > BmKn1 > BmKn2 (**Figure 2**). Therefore, when comparing the activity of peptides with varying hydrophobic and charge properties, their cumulative effects on activity should be evaluated in a systematic and comprehensive manner.

For the external membrane, the peptide’s charge is crucial for its penetration capability because of the abundance of negatively charged components like lipopolysaccharide (LPS), which can be specifically recognized and bound by the peptide (26, 27). Peptides with higher positive charges are more likely to penetrate through electrostatic interactions, thereby enhancing their penetration efficiency. Specifically, BmKn2-7, which carries six positive charges, exhibits stronger electrostatic interactions compared to BmKn1 (two positive charges) and BmKn2 (three positive charges), making it more effective in penetrating the external membrane. However, when structural differences among peptides are minimal, their antibacterial activity tends to increase with greater hydrophobicity (21). Given that BmKn1 and BmKn2 share a highly similar structure (85%), and BmKn1 exhibits a higher degree of hydrophobicity than BmKn2, BmKn1 demonstrates superior membrane penetration capability. For the bacterial inner membrane, hydrophobicity is a critical determinant of peptide penetration. Among the three peptides, BmKn1 exhibits the highest hydrophobicity, consequently demonstrating superior membrane permeability. This observation provides substantial evidence to support the conclusion.

Depolarization is often correlated with an increase in cell membrane permeability and the efflux of intracellular components, serving as a critical mechanism for peptide antibacterial activity (28, 29). This study reveals that BmKn2-7, owing to its higher charge density, exhibits the most significant depolarizing effect. This indicates a greater propensity for BmKn2-7 to form ion channels or compromise the integrity of the cell membrane, thereby leading to a more potent depolarizing action. The levels of ROS in VP treated with BmKn1, BmKn2, and BmKn2-7 were significantly elevated. Notably, the order of ROS induction paralleled the order of inner membrane penetration efficacy: BmKn1 > BmKn2-7 > BmKn2. This suggests a strong correlation between ROS production and the peptides’ ability to penetrate the bacterial inner membrane. Elevated ROS levels can induce oxidative damage to bacterial DNA, proteins, and lipids (30, 31), leading to cell death. These findings further substantiate the antibacterial activity of these peptides.

Through a comparative analysis of three peptides, it was found that the charge of the peptide played a dominant role in external membrane permeability and cell membrane depolarization, whereas hydrophobicity had a more significant impact on inner membrane permeability and ROS generation. Among the results mentioned above, BmKn1 and BmKn2-7 demonstrated superior performance compared to BmKn2. Previous studies have shown that BmKn2 exhibits notable antibacterial activity (32, 33), indicating that BmKn1 and BmKn2-7 may serve as even more effective antibacterial agents.

AMPs not only exhibit broad-spectrum antibacterial activity and high efficacy against resistant strains but also modulate the host immune system, thereby attracting significant attention for their multifaceted roles (34). In addition to directly reducing bacterial loads through their antimicrobial properties, AMPs can indirectly combat microbial invasion and infection by regulating host cellular functions (35). Specifically, they activate immune cells, enhance the production of pro-inflammatory cytokines, mitigate inflammation caused by microbes, and promote the formation of neutrophil extracellular traps (36). Wang et al. demonstrated that co-injection of antimicrobial peptides with neurotropic virus (NNV) significantly increased the survival rate of medaka compared to injection of NNV alone (37). Additionally, previous studies have shown that an antimicrobial peptide from crabs can markedly enhance the survival rate of *L. vannamei* infected with acute hepatopancreatic necrosis disease virus (38). Similarly, research on *Oncorhynchus mykiss* has found that antimicrobial peptides can effectively reduce mortality rates (39). The findings of this study demonstrate that treatment with the peptides BmKn1, BmKn2, and BmKn2-7 significantly enhanced the cumulative survival rate of *L. vannamei*, indicating that these peptides play a critical role in strengthening its immune response.

The activity of immune-related enzymes in the hepatopancreas of *L. vannamei* was investigated to elucidate their roles in the immune response. The enzyme PO is vital for immune function, catalyzing the oxidation of phenols to quinones, which polymerize into melanin, facilitating pathogen encapsulation and wound healing (5). We observed that PO activity was significantly lower in the group exposed solely to VP compared to the group co-injected with peptides and the pathogen, which exhibited markedly elevated PO activity. These results suggest that the peptides may stimulate the immune response in the hepatopancreas of shrimp, leading to increased synthesis and secretion of PO. LZM, another critical immune component, exhibits bacteriolytic activity that disrupts bacterial cell walls, thus playing a significant role in innate defense against infections (14, 40). Our findings indicate that LZM activity was significantly higher in the control group than in the other treatment groups. Therefore, it is plausible that the peptides exert an inhibitory effect on LZM activity. Additionally, the complement components C3 and C4 are essential in enhancing phagocytosis, mediating inflammation, and facilitating pathogen lysis (6). The VP group displayed the lowest levels of C3, indicating potential consumption of this component during the immune response to bacterial infection. In contrast, this group showed the highest C4 levels. The peptide injection groups exhibited opposite trends in C3 and C4 concentrations. The decline in hepatopancreatic C3 after VP injection suggests activation and subsequent consumption of C3 as part of the immune response. Conversely, the increased C4 levels may represent a compensatory mechanism, whereby the immune system enhances C4 production to strengthen the complement cascade and improve defense against the bacterial threat.

The activities of the expression of immune-related genes in the hepatopancreas of *Litopenaeus vannamei* were investigated to elucidate their roles in the immune response. *TNF-α*, *IL-1β*, and *TGF-β* genes are essential for orchestrating a balanced immune response, contributing to both the defense against pathogens and the maintenance of immune homeostasis in *L.vannamei* (41–43). In this experiment, following infection with VP, a significant upregulation of the pro-inflammatory cytokine TNF-α and a concurrent downregulation of the anti-inflammatory cytokine TGF-β in the *L. vannamei* were observed. These alterations suggest that an inflammatory response has been activated in the hepatopancreas at this stage. The mRNA expression levels of *TNF-α* and *IL-1β* were significantly decreased following peptide supplementation, indicating that these peptides can effectively attenuate the inflammatory response elicited by *Vibrio parahaemolyticus* infection. Administration of antibacterial peptides to shrimp infected with VP rapidly inhibited bacterial proliferation and reproduction, effectively reducing pathogen load and thereby alleviating the burden on the immune system.

*ALF*, *Crus*, and *Pen-3* genes encode peptides that are critical components of the shrimp’s immune system, forming a multi-layered defense mechanism against diverse pathogens. The upregulation of their expression in response to infection underscores their vital role in sustaining health and survival in the pathogen-rich environments typical of shrimp habitats (44, 45). In the research, the relative expression levels of the ALF and Crus genes were markedly increased in the VP group in comparison to the groups receiving peptide injections. This elevation is likely attributable to the activation of the innate immune response as a direct defense mechanism against bacterial infection. Conversely, the diminished expression of these genes in the co-injection group (peptides and VP) may be attributed to the antimicrobial properties of the peptides. The introduction of these antimicrobial peptides likely provides external support to the shrimp’s immune system, thereby reducing the reliance on endogenous production of *ALF* and *Crus* as primary defense mechanisms. This supplementation may modulate the shrimp’s immune response by partially alleviating the burden on the internal immune machinery for producing these peptides, leading to the observed reduction in their gene expression.

Environmental or physiological stressors trigger the cellular apoptosis pathway, which is a critical response mechanism that enables shrimp to mitigate cellular damage and preserve overall health under adverse conditions (46). In *L. vannamei*, genes such as *Cyt-c*, *Bax*, *Bcl-2*, *Caspase-3*, *Caspase-8*, and *P53* play pivotal roles in regulating cellular apoptosis (47–49). Previous research has demonstrated that six hours post-infection with *Vibrio alginolyticus*, there is a significant upregulation of apoptosis-related genes in both the hemocytes and hepatopancreas of *L. vannamei* (50). In this study, we observed that the relative expression levels of apoptosis-related genes were significantly higher in the VP group compared to the peptide injection groups. This difference is likely attributable to the host’s cellular response to bacterial infection. The pathogenic bacteria induce intracellular stress and damage, thereby activating apoptosis pathways as a defensive mechanism to contain and limit the spread of infection through the elimination of infected cells. In contrast, the downregulation of apoptosis-related genes observed in the group co-injected with peptides and VP indicates a protective role of the peptides. These peptides may alleviate cellular stress and damage caused by bacterial infection, potentially through direct inhibition of bacterial activity or by enhancing the shrimp’s overall immune response.

Furthermore, this study investigated the effects of *Vibrio parahaemolyticus* and BmKn2-7 on *L. vannamei vannamei* through intestinal transcriptome analysis. The results showed that in the CG-vs-VP comparison group, 320 genes were upregulated and 804 genes were downregulated; in the CG-vs-BmKn2-7 comparison group, 239 genes were upregulated and 1,711 genes were downregulated; and in the VP-vs-BmKn2-7 comparison group, 310 genes were upregulated and 382 genes were downregulated. GO enrichment analysis revealed that numerous GO terms related to immunity were identified in the CG-vs-VP comparison group. In the CG-vs-VP comparison group, evasion or tolerance of host defense response was found to be the most significantly enriched. This may be related to the interference of *Vibrio parahaemolyticus* with the host’s immune signaling pathways. *V. parahaemolyticus* can secrete certain toxic factors or signaling molecules that disrupt the immune signaling pathways of shrimp (51). For example, specific proteases produced by *V. parahaemolyticus* can degrade key proteins involved in the immune signal transduction process in shrimp, preventing proper transmission of immune signals (52). Consequently, immune cells fail to be effectively activated, resulting in the inability to initiate a robust immune response to eliminate the pathogen. The previous study revealed that *V. parahaemolyticus* disrupts the antioxidative system in *Penaeus monodon*, inducing oxidative stress and tissue damage through altered activities of antioxidant enzymes (53). This study also found that oxidoreductase activity was significantly enriched, indicating that *Vibrio parahaemolyticus* infection induces oxidative stress in host tissues, resulting in dysregulation of redox-related gene expression and further impairing the host’s immune response. The immune system of *Litopenaeus vannamei* relies on innate immunity and lacks specific adaptive immune responses (54). Chitin-binding proteins play a critical role in the shrimp’s immune defense. In the immune systems of crustaceans such as shrimp, as well as in insects, chitin-binding proteins function as opsonins by binding to pathogens, thereby promoting the phagocytosis of pathogens by phagocytic cells (55). Moreover, chitin-binding proteins can activate the complement system or other immune signaling pathways, triggering a series of immune responses that further enhance the organism’s ability to eliminate pathogens (56). These findings underscore their important role in pathogen recognition and immune defense. In this study, the VP-vs-BmKn2-7 comparison group showed significant enrichment of chitin binding, which may be attributed to IsCT’s ability to suppress overactivated immune responses, allowing immune cells and effector molecules to return to homeostasis. This, in turn, reduces the expression levels of genes associated with oxidative stress and pathogen recognition. The Peroxisome Proliferator-Activated Receptor (PPAR) signaling pathway is mediated by a family of nuclear receptor members (PPARs) and is widely present in animals, regulating various physiological processes such as metabolism, inflammation, immunity, and cell differentiation (57). The PPAR family mainly includes three subtypes: PPAR-α, PPAR-β/δ, and PPAR-γ. PPAR-α and PPAR-γ reduce the production of pro-inflammatory factors by downregulating inflammatory pathways such as NF-κB and AP-1 (58). Additionally, PPAR-γ promotes macrophage polarization from the pro-inflammatory M1 phenotype to the anti-inflammatory M2 phenotype, thereby mitigating inflammation (59). In this study, the VP-vs-BmKn2-7 comparison group showed significant enrichment of PPAR-related terms, which may be attributed to the effects of IsCT in modulating the PPAR signaling pathway to alleviate inflammation, mitigate oxidative stress, and restore metabolic homeostasis.

Liu et al. demonstrated that transcriptome analysis of the hepatopancreas in *Neocaridina denticulata* after *Vibrio parahaemolyticus* infection revealed that numerous DEGs were enriched in immune-related pathways (60). A similar study has also been reported in Sparus macrocephalus (61). The results of this study also revealed significant enrichment of immune-related pathways following *Vibrio parahaemolyticus* infection, but the largest number of DEGs were annotated to metabolic pathways. The PCK2 gene plays a crucial role in regulating gluconeogenesis and energy metabolism, contributing to environmental adaptation, immune defense, oxidative stress alleviation, and reproductive development in the organism (62). PCK2 may help maintain the balance of the host immune system during inflammation and infection by modulating energy metabolism, thereby preventing excessive immune responses from causing damage to the host (63). By enhancing energy supply, PCK2 supports cells in combating oxidative stress, maintaining mitochondrial function, and preserving cellular homeostasis (64). In this study, the VP-vs-BmKn2-7 comparison group revealed that the *PCK2* gene was annotated in numerous significantly enriched pathways, indicating that *PCK2* plays a critical role in immune regulation. The FoxO signaling pathway is involved in the differentiation and functional regulation of immune cells, such as T cells, and mitigates chronic inflammation by suppressing the NF-κB signaling pathway (65). However, under certain conditions, it may also promote the release of inflammatory cytokines. Additionally, FoxO can enhance cellular resistance to oxidative stress by inducing the expression of antioxidant enzymes, such as superoxide dismutase and catalase (66). AMPK activation can inhibit the transcriptional activity of NF-κB, reduce the production of pro-inflammatory cytokines such as TNF-α and IL-6, and downregulate inflammation mediated by Toll-like receptors (67). In macrophages, AMPK modulates the activity of the NLRP3 inflammasome, thereby suppressing caspase-1-dependent pyroptosis and the release of IL-1β (68). Leukocyte transendothelial migration is a central process in immune responses, regulating the migration of leukocytes from the vascular lumen to inflamed or infected tissues, thereby coordinating innate and adaptive immunity. Its functions span multiple aspects, including immune defense, inflammation regulation, and tissue repair (69). The three immune-related signaling pathways were significantly enriched in the VP-vs-BmKn2-7 comparison group in this study. This enrichment may result from the combined effects of antimicrobial peptides in enhancing immune regulation, alleviating inflammation and oxidative stress, optimizing metabolism, and supporting immune cell migration.

In summary, the *in vitro* mechanisms of membrane permeabilization and ROS elevation induced by the three peptides likely underpin their *in vivo* antibacterial efficacy and immunomodulatory effects. Enhanced membrane permeability directly compromises bacterial membrane integrity, thereby reducing pathogen load and infection severity. Concurrently, elevated ROS levels not only mediate oxidative damage to pathogens but also modulate innate immune responses through the activation of pro-inflammatory signaling pathways and by influencing mitochondrial function, which may promote antigen presentation and immune cell activation.

## 5 Conclusions

In conclusion, the functional characterization of BmKn1 has been successfully elucidated. The properties and test results of BmKn1, BmKn2, and BmKn2-7, derived from scorpion venom, demonstrate significant potential as antimicrobial agents against *Vibrio parahaemolyticus* (VP) and as immune enhancers for *Litopenaeus vannamei*. The application of these peptides is expected to promote the development and advancement of the *L. vannamei* aquaculture industry.

## Data Availability

The datasets presented in this study can be found in online repositories. The names of the repository/repositories and accession number(s) can be found in the article/**Supplementary Material**.
