# Supplementary material for: Scorpion venom peptides enhance immunity and survival in Litopenaeus vannamei through antibacterial action against Vibrio parahaemolyticus
Source: Front Immunol. 2025 Apr 22;16:1551816. doi: 10.3389/fimmu.2025.1551816 (PMC12053305; doi:10.3389/fimmu.2025.1551816)
Supplement: Supplementary file 1 [file SupplementaryFile1.docx]

**1. Intestinal microbiota analysis**

**1.1 Quality control and clustering**

**1.1.1 Reads filtering**

Raw data containing adapters or low-quality reads would affect the followinganalysi. Thus, to get high quality clean reads, raw reads were further filtered according to the following rules using FASTP (Chen et al., 2018) (version 0.18.0): ①Removing reads containing more than 10% of unknown nucleotides (N); ② Removing reads containing more than 50% of bases with quality (Q-value) < 20. ③ Removing adapter contamination.

**1.1.2 Reads Merging**

Paired reads were overlaped as raw tags using FLASH (Magoc et al., 2011) (version 1.2.11) with a minimum overlap of 10 bp and mismatch error rates of 2%.

**1.1.3 Raw tag filtering**

Noisy sequences of raw tags were filtered under specific filtering conditions (Bokulich et al., 2013) to obtain the high-quality clean tags. The filtering conditions are as follows：① Break raw tags from the first low quality base site where the number of bases in the continuous low quality value (the default quality threshold is ≤3) reaches the set length (the default length is 3 bp)；② Then, filter tags whose continuous high-quality base length is less than 75% of the tag length.

**1.1.4 Clustering and chimera removal**

The clean tags were clustered into operational taxonomic units (OTUs) of ≥ 97 % similarity using UPARSE (Edgar et al., 2013) (version 9.2.64) pipeline. All chimeric tags were removed using UCHIME algorithm (Edgar et al., 2011) and finally obtained effective tags for further analysis. The tag sequence with highest abundance was selected as representative sequence within each cluster.

**1.2 Taxonomy annotation**

The representative OTU sequences or ASV sequences were classified into organisms by a naïve Bayesian model using RDP classifier (Wang et al., 2007) (version 2.2) based on SILVA database (Pruesse et al., 2007) (version 138.1) or UNITE database (Nilsson et al., 2019) (version 8.3), with the confidence threshold value of 0.8.

**1.3 Community composition analysis**

The abundance statistics of each taxonomy was visualized using Krona (Ondov et al., 2011) (version 2.6). The stacked bar plot of the community composition was visualized in R project ggplot2 package (version 2.2.1). Circular layout representations of species abundance were graphed using circus (Krzywinski et al., 2009) (version 0.69-3). Heatmap of species abundance was plotted using pheatmap package (version 1.0.12) in R project. Pearson correlation analysis of species was calculated in R project psych package (Revelle et al., 2015) (version 1.8.4). Network of correlation coefficient were generated using Omicsmart, a dynamic real-time interactive online platform for data analysis (http://www.omicsmart.com) or igraph package (Csardi et al., 2013) (version 1.1.2) in R project.

1.4 Indicator species analysis

Between groups Venn analysis was performed in R project VennDiagram package (Chen et al., 2011) (version1.6.16) and upset plot was performed in R project UpSetR package (Dixon et al., 2003) (version 1.3.3) to identify unique and common species or OTUs or ASVs. Species comparison between groups was calculated by welch's t-test and wilcoxon rank test in R project Vegan package (Wang et al., 2007) (version 2.5.3). Species comparison among groups was computed by tukey’s HSD test and kruskal-wallis H test in R project Vegan package (Dixon et al., 2003) (version 2.5.3). Biomarker features in each group were screened by LEfSe software (Segata et al., 2011) (version 1.0), randomforest package (Cerdó et al., 2024) (version 4.6.12) in R project, pROC package (Robin et al., 2011) (version 1.10.0) in R project, and labdsv package (version2.0-1) in R project. Ternary plot of species abundance was plotted using R ggtern package (Hamilton et al., 2018) (version 3.1.0).

**1.5 Alpha diversity analysis**

Chao1, ACE, Shannon, Simpson, Good’s coverage, Pielou’s evenness index were calculated in QIIME (Caporaso et al., 2010) (version 1.9.1). PD-whole tree index was calculated in picante (Kembel et al., 2010) (version 1.8.2). OTU/ASV rarefaction curve and rank abundance curves were plotted in R project ggplot2 package (version 2.2.1). Alpha index comparison between groups was calculated by Welch's t-test and Wilcoxon rank test in R project Vegan package (Dixon et al., 2003) (version 2.5.3). Alpha index comparison among groups was computed by Tukey’s HSD test and Kruskal-Wallis H test in R project Vegan package (Dixon et al., 2003) (version 2.5.3).

**1.6 Beta diversity analysis**

Sequence alignment was performed using Muscle (Edgar et al., 2004) (version 3.8.31) and phylogenetic tree was constructed using FastTree (Price et al., 2010) (version 2.1), then weighted and unweighted unifrac distance matrix were generated by GuniFrac package (Lozupone et al., 2005) (version 1.0) in R project. Jaccard and bray-curtis distance matrix calculated in R project Vegan package (Dixon et al., 2003) (version 2.5.3). PCA (principal component analysis) was performed in R project Vegan package (Dixon et al., 2003) (version 2.5.3). Multivariate statistical techniques including PCoA (principal coordinates analysis) and NMDS (non-metric multi-dimensional scaling) of (Un) weighted unifrac, jaccard and bray-curtis distances were

generated in R project Vegan package (Dixon et al., 2003) (version 2.5.3) and plotted in R project ggplot2 package (version 2.2.1). Statistic analysis of Welch’s t-test, Wilcoxon rank test, Tukey’s HSD test, Kruskal-Wallis H test, Adonis (also called Permanova) and Anosim test was calculated in R project Vegan package (Dixon et al., 2003) (version 2.5.3).

Bokulich, N. A., Subramanian, S., Faith, J. J., Gevers, D., Gordon, J. I., Knight, R., Caporaso, J. G., 2013. Quality-filtering vastly improves diversity estimates from illumina amplicon sequencing. Nature Methods, 10(1), 57-U11. https:// doi.org /:10.1038/nmeth.2276.

Cerdó, T., & Moral, T. T., 2024. Potential risk genes for primary sjogren's syndrome from a meta-analysis by linear regression and random forest classification. Genes. Dis. 11(3). https:// doi.org /: 1 0. 10 16/j.gendis.2023.05.015.

Chen, H., & Boutros, P. C., 2011. Venndiagram: A package for the generation of highly-customizable venn and euler diagrams in r. Bmc Bioinformatics, 12. https:// doi.org /:10.1186/1471-2105-12-35.

Chen, S. F., Zhou, Y. Q., Chen, Y. R., Gu, J., 2018. Fastp: An ultra-fast all-in-one fastq preprocessor. Bioinformatics, 34(17), 884-890. https:// doi.org /:10.1093/bioinformatics/bty560.

Dixon, P., 2003. Vegan, a package of r functions for community ecology. J. Veg. Sci. 14(6), 927-930. https:// doi.org /:10.1658/1100-9233(2003)014[0927:Vaporf]2.0.Co;2.

Edgar, R. C., 2013. Uparse: Highly accurate otu sequences from microbial amplicon reads. Nature Methods, 10(10), 996. https:// doi.org /:10.1038/nmeth.2604.

Edgar, R. C., Haas, B. J., Clemente, J. C., Quince, C., & Knight, R., 2011. Uchime improves sensitivity and speed of chimera detection. Bioinformatics. 27(16), 2194-2200. https:// doi.org /:10.1093/bioinformatics/btr381.

Hamilton, N. E., & Ferry, M., 2018. Ggtern: Ternary diagrams using ggplot2. J. Stat. Softw. 87(CN3), 1-17. https:// doi.org /:10.18637/jss.v087.c03.

Kembel, S. W., Cowan, P. D., Helmus, M. R., Cornwell, W. K., Morlon, H., Ackerly, D. D., Webb, C. O., 2010. Picante: R tools for integrating phylogenies and ecology. Bioinformatics, 26(11), 1463-1464. https:// doi.org /:10.1093/bioinformatics/btq166.

Krzywinski, M., Schein, J., Birol, I., Connors, J., Gascoyne, R., Horsman, D., Marra, M. A., 2009. Circos: An information aesthetic for comparative genomics. Genome Res. 19(9), 1639-1645. https:// doi.org /:10.1101/gr.092759.109.

Lozupone, C., & Knight, R., 2005. Unifrac: A new phylogenetic method for comparing microbial communities. Appl. Environ. Microb. 71(12), 8228-8235. https:// doi.org /:10.1128/aem.71.12.8228-8235.2005.

Magoc, T., & Salzberg, S. L., 2011. Flash: Fast length adjustment of short reads to improve genome assemblies. Bioinformatics. 27(21), 2957-2963. https:// doi.org /:10.1093/bioinformatics/btr507

Nilsson, R. H., Larsson, K. H., Taylor, A. F. S., Bengtsson-Palme, J., Jeppesen, T. S., Schigel, D., Abarenkov, K., 2019. The unite database for molecular identification of fungi: Handling dark taxa and parallel taxonomic classifications. Nucleic Acids Res. 47(D1), D259-D264. https:// doi.org /:10.1093/nar/gky1022.

Ondov, B. D., Bergman, N. H., & Phillippy, A. M., 2011. Interactive metagenomic visualization in a web browser. Bmc Bioinformatics, 12. https:// doi.org /:10.1186/1471-2105-12-385.

Price, M. N., Dehal, P. S., & Arkin, A. P., 2010. Fasttree 2-approximately maximum-likelihood trees for large alignments. Plos One, 5(3). https:// doi.org /:10.1371/journal.pone.0009490.

Pruesse, E., Quast, C., Knittel, K., Fuchs, B. M., Ludwig, W. G., Peplies, J., & Glöckner, F. O., 2007. Silva: A comprehensive online resource for quality checked and aligned ribosomal rna sequence data compatible with arb. Nucleic Acids Res. 35(21), 7188-7196. https:// doi.org /:10.1093/nar/gkm864.

Robin, X., Turck, N., Hainard, A., Tiberti, N., Lisacek, F., Sanchez, J. C., & Müller, M., 2011. Proc: An open-source package for r and s plus to analyze and compare roc curves. Bmc Bioinformatics, 12. https:// doi.org /:10.1186/1471-2105-12-77.

Segata, N., Izard, J., Waldron, L., Gevers, D., Miropolsky, L., Garrett, W. S., & Huttenhower, C., 2011. Metagenomic biomarker discovery and explanation. Genome Biol. 12(6). https:// doi.org /:10.1186/gb-2011-12-6-r60.

Wang, Q., Garrity, G. M., Tiedje, J. M., & Cole, J. R., 2007. Naive bayesian classifier for rapid assignment of rRNA sequences into the new bacterial taxonomy. Appl. Environ. Microb. 73(16), 5261-5267. https:// doi.org /:10.1128/aem.00062-07.

**2. Transcriptome sequencing analysis**

**2.1 RNA Extraction, library construction and sequencing**

Total RNA was extracted using Trizol reagent kit (Invitrogen, Carlsbad, CA, USA) according to the manufacturer’s protocol. RNA quality was assessed on an Agilent 2100 Bioanalyzer (Agilent Technologies, Palo Alto, CA, USA) and checked using RNase free agarose gel electrophoresis. After total RNA was extracted, eukaryotic mRNA was enriched by Oligo(dT) beads. Then the enriched mRNA was fragmented into short fragments using fragmentation buffer and reversly transcribed into cDNA by using NEBNext Ultra RNA Library Prep Kit for Illumina (NEB #7530, New England Biolabs, Ipswich, MA, USA). The purified double-stranded cDNA fragments were end repaired, A base added, and ligated to Illumina sequencing adapters. The ligation reaction was purified with the AMPure XP Beads (1.0X). And polymerase chain reaction (PCR) amplified.The resulting cDNA library was sequenced using Illumina Novaseq6000 by Gene Denovo Biotechnology Co. (Guangzhou, China).

**2.2 Bioinformatics analysis**

**2.2.1 Filtering of Clean Reads**

Reads obtained from the sequencing machines includes raw reads containing adapters or low-quality bases which will affect the following assembly and analysis. Thus, to get high quality clean reads, reads were further filtered by fastp [1] (version 0.18.0). The parameters were as follows: 1) removing reads containing adapters; 2) removing reads containing more than 10% of unknown nucleotides(N); 3) removing low quality reads containing more than 50% of low quality (Q-value≤20) bases.

**2.2.2 Alignment with Ribosome RNA (rRNA)**

Short reads alignment tool Bowtie2 [2] (version 2.2.8) was used for mapping reads to ribosome RNA (rRNA) database. The rRNA mapped reads then will be removed. The remaining clean reads were further used in assembly and gene abundance calculation.

**2.2.3 Alignment with Reference Genome**

An index of the reference genome was built, and paired-end clean reads were mapped to the reference genome using HISAT2. 2.4[3] and other parameters set as a default.

**2.2.4 Quantification of Gene Abundance**

The mapped reads of each sample were assembled by using StringTie v1.3.1[4][5] in a reference-based approach. For each transcription region, a FPKM (fragment per kilobase of transcript per million mapped reads) value was calculated to quantify its expression abundance and variations, using RSEM [6] software.

**2.3 Relationship analysis of samples**

**2.3.1 Correlation Analysis of Replicas**

Correlation analysis was performed by R. Correlation of two parallel experiments provides the evaluation of the reliability of experimental results as well as operational stability. The correlation coefficient between two replicas was calculated to evaluate repeatability between samples. The closer the correlation coefficient gets to 1, the better the repeatability between two parallel experiments.

**2.3.2 Principal Component Analysis**

Principal component analysis (PCA) was performed with R package gmodels (http://www.r-project.org/) in this experience. PCA is a statistical procedure that converts hundreds of thousands of correlated variables (gene expression) into a set of values of linearly uncorrelated variables called principal components. PCA is largely used to reveal the structure/relationship of the samples/datas.

**2.4 Differentially expressed genes (DEGs)**

RNAs differential expression analysis was performed by DESeq2[7] software between two different groups (and by edgeR[8] between two samples). The genes/transcripts with the parameter of false discovery rate (FDR) below 0.05 and absolute fold change≥2 were considered differentially expressed genes/transcripts.

**2.4.1 GO Enrichment Analysis**

Gene Ontology (GO) [9] is an international standardized gene functional classification system which offers a dynamic-updated controlled vocabulary and a strictly defined concept to comprehensively describe properties of genes and their products in any organism. GO has three ontologies: molecular function, cellular component and biological process. The basic unit of GO is GO-term. Each GO-term belongs to a type of ontology. GO enrichment analysis provides all GO terms that significantly enriched in DEGs comparing to the genome background, and filter the DEGs that correspond to biological functions. Firstly, all DEGs were mapped to GO terms in the Gene Ontology database (http://www.geneontology.org/), gene numbers were calculated for every term, significantly enriched GO terms in DEGs comparing to the genome background were defined by hypergeometric test.

**2.4.2 Pathway Enrichment Analysis**

Genes usually interact with each other to play roles in certain biological functions. Pathway-based analysis helps to further understand genes biological functions. KEGG [10] is the major public pathway-related database. Pathway enrichment analysis identified significantly enriched metabolic pathways or signal transduction pathways in DEGs

comparing with the whole genome background.

**2.4.3 Disease Ontology Enrichment Analysis (only apply in humangenome reference)**

The Disease Ontology (DO) [11] has been developed as a standardized ontology for human disease with the purpose of providing the biomedical community with consistent, reusable and sustainable descriptions of human disease terms, phenotype characteristics and related medical vocabulary disease concepts. DO enrichment analysis identified significantly enriched human disease DO terms in DEGs comparing with the whole genome background.

**2.4.4 Reactome Enrichment Analysis (only apply in human genome reference)**

The Reactome[12][13] is a free online database of biological pathways. The core unit of the Reactome data model is the reaction. Entities (nucleic acids, proteins, complexes and small molecules) participating in reactions form a network of biological interactions andare grouped into pathways. Examples of biological pathways in Reactome include signaling, innate and acquired immune function, transcriptional regulation, translation, apoptosis and classical intermediary metabolism.

**2.5 Gene Set Enrichment Analysis (GSEA)**

We performed gene set enrichment analysis using software GSEA [14] and MSigDB[14] to identify whether a set ofgenes in specific GO terms\KEGG pathways\Reactome pathways\DO terms shows significant differences in two groups. Briefly, we input geneexpression matrix and rank genes by SignaltoNoise normalization method. Enrichment scores and *p* value was calculated in default parameters.

**2.6 Protein-Protein interaction**

Protein-Protein interaction network was identified using String v10[15], which determined genes as nodes and interaction as lines in a network. The network file was visualized using Cytoscape (v3.7.1) [16] software to present a core and hub gene biological interact.

**2.7 Gene Structure Optimization (not available in human reference)**

The gene structure and annotation for model organisms such as people, mice and Arabidopsis thaliana are almost complete, but for other species, the reads can be used for optimizing their gene structure, thus perfecting their gene annotations. After mapping reads to reference genome, the Hisat2 was used in reconstruction of transcripts which may extend the 5' untranslated region (5’UTR) or 3’UTR of gene to optimize the gene structure.

**2.8 Single-nucleotide Polymorphism (SNP) Analysis**

The bcftools [17] was used for calling variants of transcripts, and ANNOVAR was used for SNP/InDel annotation. The function, genome site and type of variation ofSNPs were also analyzed.

**2.9 RNA editing**

Use the following criteria to screen reliable editing sites from SNP sites [18] [19]: (1) Removing the low-quality SNPs while calling SNP by bcftools. (2) Correcting the SNPs around InDel region. (3) Choosing non-overlapping SNPs in UTR and EXONregion. (4) Choosing SNPs with reference reads>=2 and variate reads>=3. (5) Choosing SNPs with the mutation frequency between 0.1 and 0.9.

**2.10 Alternative Splicing Analysis**

The software rMATS [20] (version 4.0.1) (http://rnaseq-mats.sourceforge.net/index.html) was used to identify alternative splicing events and analyze differential alternative splicing events between samples. We identified AS events with a false discovery rate (FDR) <0.05 in a comparison as significant AS events. The classification of alternative splicing is as follows: (1) SE: skipped exon. (2) MXE: mutually exclusive exon. (3) A5SS: alternative 5’ splicesite. (4) A3SS: alternative 3’ splicesite. (5) RI: retained intron.

**Reference**

[1] Chen S, Zhou Y,Chen Y,et al. fastp: an ultra-fast all-in-one FASTQ preprocessor[J]. bioRxiv, 2018: 274100.

[2] Langmead B, Salzberg S L. Fast gapped-read alignment with Bowtie 2[J]. Nature methods, 2012, 9(4): 357-359.

[3] Kim D, Langmead B, Salzberg S L. HISAT: a fast spliced aligner with low memory requirements[J]. Nature methods, 2015, 12(4): 357.

[4] Pertea M, Pertea G M, Antonescu C M, et al. StringTie enables improved reconstruction of a transcriptome from RNA-seq reads[J]. Nature biotechnology, 2015, 33(3): 290.

[5] Pertea M, Kim D, Pertea G M, et al. Transcript-level expression analysis ofRNA-seq experiments with HISAT, StringTie and Ballgown[J]. Nature protocols, 2016, 11(9): 1650.

[6] Li B, Dewey CN. RSEM: accurate transcript quantification from RNA-Seq data with or without a reference genome. BMC Bioinformatics. 2011; 12:323. Published 2011 Aug 4. doi:10.1186/1471-2105-12-323

[7] Love M I, Huber W, Anders S. Moderated estimation of fold change and dispersion for RNA-seq data with DESeq2[J]. Genome biology, 2014, 15(12): 550.

[8] Robinson MD, McCarthy D J, Smyth G K. edgeR: a Bioconductor package for differential expression analysis of digital gene expression data[J]. Bioinformatics, 2010, 26(1): 139-140.

[9] Ashburner M, Ball C A, Blake J A, et al. Gene ontology: tool for the unification of biology[J]. Nature genetics, 2000, 25(1): 25.

[10] Kanehisa M, Goto S. KEGG: kyoto encyclopedia of genes and genomes[J]. Nucleic acids research, 2000, 28(1): 27-30.

[11] Schriml L M, Arze C, Nadendla S, et al. Disease Ontology: a backbone for disease semantic integration[J]. Nucleic acids research, 2011, 40(D1): D940-D946.

[12] Croft D, O’kelly G, Wu G, et al. Reactome: a database of reactions, pathways and biological processes[J]. Nucleic acids research, 2010, 39(suppl_1): D691-D697.

[13] Fabregat A, Jupe S, Matthews L, et al. The reactome pathway knowledgebase[J]. Nucleic acids research, 2017, 46(D1): D649-D655.

[14] Subramanian A, Tamayo P, Mootha V K, et al. Gene set enrichment analysis: a knowledge-based approach for interpreting genome-wide expression profiles[J]. Proceedings of the National Academy of Sciences, 2005, 102(43): 15545-15550.

[15] Szklarczyk D, Franceschini A, Wyder S, et al. STRING v10: protein–protein interaction networks, integrated over the tree of life[J]. Nucleic acids research, 2014, 43(D1): D447-D452.

[16] Shannon P, Markiel A, Ozier O, et al. Cytoscape: a software environment for integrated models of biomolecular interaction networks[J]. Genome research, 2003, 13(11):2498-2504.

[17] Arthur, W, Pightling, et al. Choice of reference-guided sequence assembler and SNP caller for analysis of Listeria monocytogenes short-read sequence data greatly influences rates of error[J]. Bmc Research Notes, 2015, 8(1): 1-9.

[18] Gokul R., Rui Z., Robert P. Identifying RNA editing sites using RNA sequencing data alone, Nat Methods. 2013 February; 10(2): 128–132.

[19] Jae Hoon B., Jae-Hyung L., Gang Li, Accurate identification of A-to-I RNA editing in human by transcriptome sequencing, Genome Res. 2012 22:142-150.

[20] Shen S, Park J W, Lu Z, et al. rMATS: robust and flexible detection of differential alternative splicing from replicate RNA-Seq data[J]. Proceedings of the National Academy of Sciences, 2014, 111(51): E5593-E5601.

**
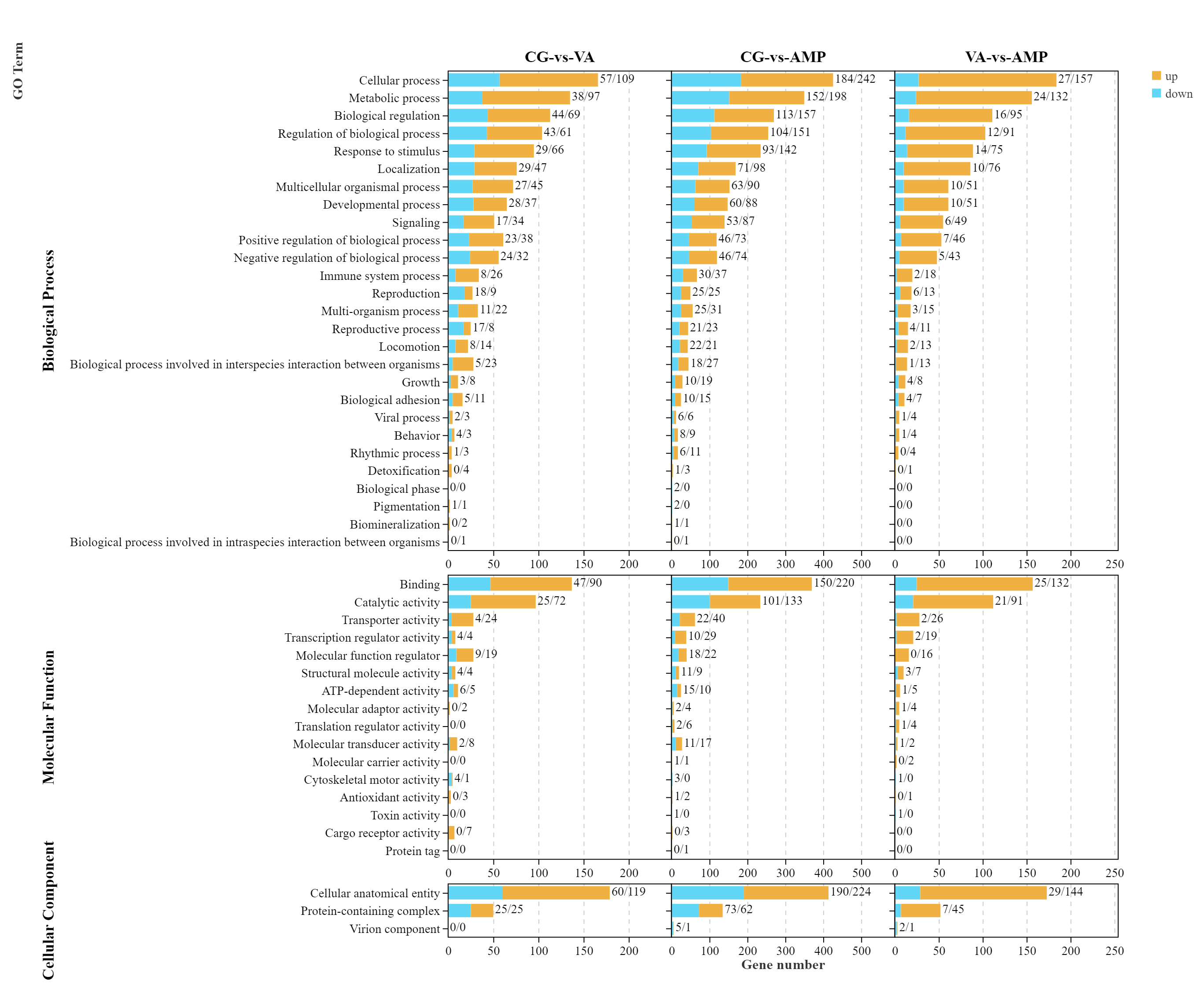
**

Fig. S1 Statistical plot of GO annotated classification of different comparison groups. Yellow histograms represent up-regulated genes and blue histograms represent down-regulated genes.

**
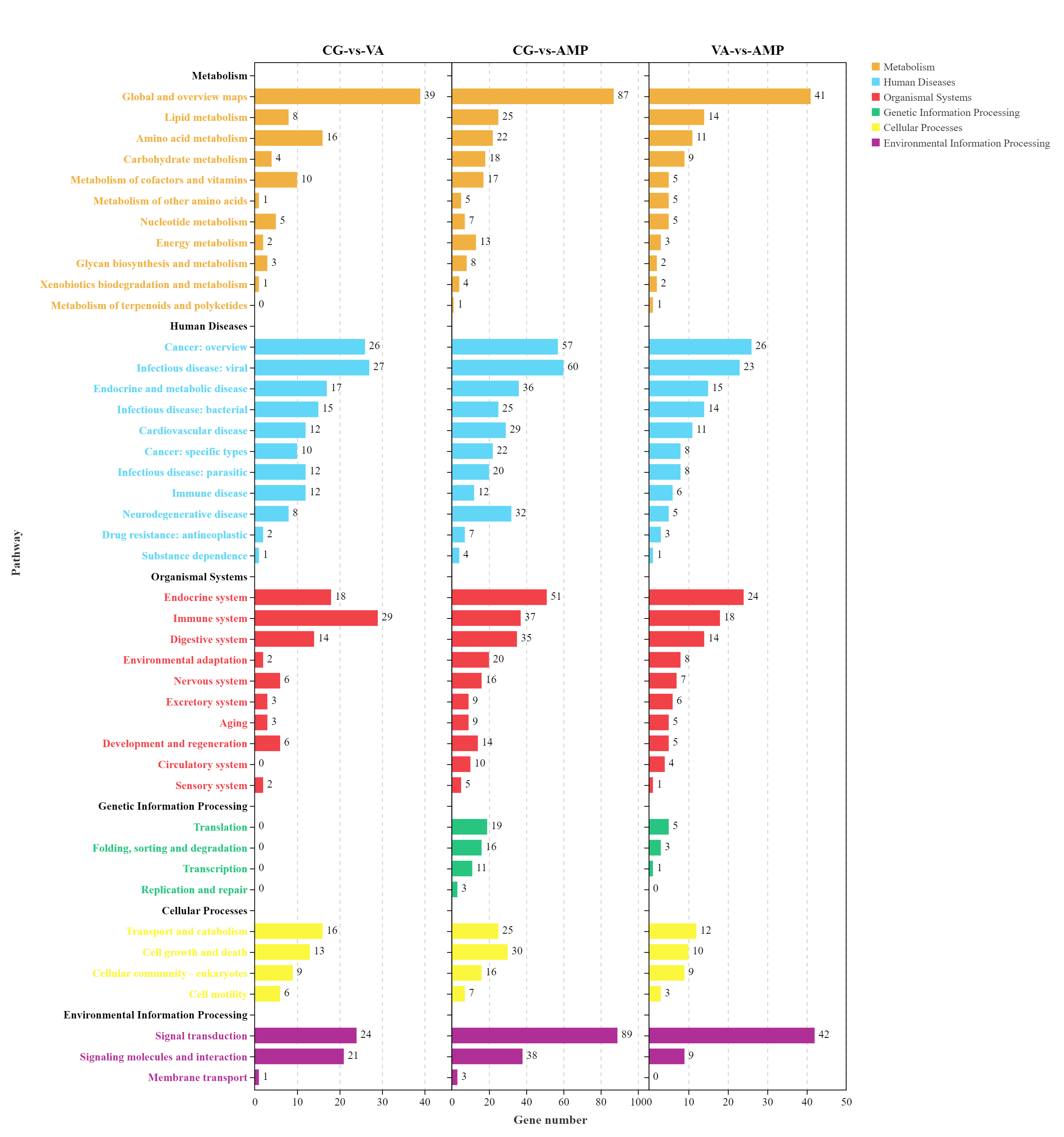
**

Fig. S2 Statistical plot of KEGG metabolic pathway classification in different comparison groups.
